# Acetate Revisited: A Key Biomolecule at the Nexus of Metabolism, Epigenetics, and Oncogenesis – Part 2: Acetate and ACSS2 in Health and Disease

**DOI:** 10.3389/fphys.2020.580171

**Published:** 2020-11-12

**Authors:** John R. Moffett, Narayanan Puthillathu, Ranjini Vengilote, Diane M. Jaworski, Aryan M. Namboodiri

**Affiliations:** ^1^ Department of Anatomy, Physiology and Genetics, and Neuroscience Program, Uniformed Services University of the Health Sciences, Bethesda, MD, United States; ^2^ Department of Neurological Sciences, University of Vermont College of Medicine, Burlington, VT, United States

**Keywords:** acetyl coenzyme A, acetyl-CoA synthetase, aspartoacylase, microbiome, acyl-CoA short-chain synthetase, N-acetylaspartate, NAT8L, glyceryl triacetate

## Abstract

Acetate, the shortest chain fatty acid, has been implicated in providing health benefits whether it is derived from the diet or is generated from microbial fermentation of fiber in the gut. These health benefits range widely from improved cardiac function to enhanced red blood cell generation and memory formation. Understanding how acetate could influence so many disparate biological functions is now an area of intensive research. Protein acetylation is one of the most common post-translational modifications and increased systemic acetate strongly drives protein acetylation. By virtue of acetylation impacting the activity of virtually every class of protein, acetate driven alterations in signaling and gene transcription have been associated with several common human diseases, including cancer. In part 2 of this review, we will focus on some of the roles that acetate plays in health and human disease. The acetate-activating enzyme acyl-CoA short-chain synthetase family member 2 (ACSS2) will be a major part of that focus due to its role in targeted protein acetylation reactions that can regulate central metabolism and stress responses. ACSS2 is the only known enzyme that can recycle acetate derived from deacetylation reactions in the cytoplasm and nucleus of cells, including both protein and metabolite deacetylation reactions. As such, ACSS2 can recycle acetate derived from histone deacetylase reactions as well as protein deacetylation reactions mediated by sirtuins, among many others. Notably, ACSS2 can activate acetate released from acetylated metabolites including N-acetylaspartate (NAA), the most concentrated acetylated metabolite in the human brain. NAA has been associated with the metabolic reprograming of cancer cells, where ACSS2 also plays a role. Here, we discuss the context-specific roles that acetate can play in health and disease.

## Introduction

Diabetes, obesity, heart disease, cancer, and liver disease have all been linked in various ways to acetate availability, metabolism, and signaling. Acetate supplementation induces physiological and biochemical responses including inhibition of insulin release, weight loss, cardiac protection, anti-inflammatory actions, protein acetylation, apoptosis of cancer cells, erythropoiesis signaling, and enhancement of chemotherapeutic glioma treatment, among many others. Although circulating acetate levels are lower in humans than in most mammalian species, dietary acetate, and acetate supplementation can exert positive effects on human health. Indeed, the health benefits and medical uses of vinegar, the predominant constituent of which is acetic acid, have been claimed since antiquity (Johnston and Gaas, 2006; Budak et al., 2014). Vinegar provided the first insights on the impact of short-chain fatty acids in nutrition and health.

In contrast to the documented health benefits of acetate supplementation and acetic acid in the diet, acetate signaling and metabolism have also been linked to disease states, such as cancer. One focus of cancer research involves the metabolic resources for biomass accumulation. Glucose and glutamine are some of the primary metabolites funneled into the citric acid cycle of cancer cells for energy derivation to support the abundant macromolecular biosynthesis required for tumorigenesis. Acetate is strongly implicated as another key molecule facilitating biomass accumulation in rapidly dividing cancer cells. Additional metabolites that provide substrate for biosynthetic pathways include fatty acids, lactate, and N-acetylaspartate (NAA). In the second part of this review, we will focus on acetate metabolism as it relates to human health, as well as the possible therapeutic potential of acetate supplementation. This will highlight the multiple roles that acetate plays in transcriptional regulation and cancer metabolism.

Indeed, acetate has acquired somewhat of a dual reputation in recent years. On the one hand, it has long been considered a healthy dietary constituent and potential treatment for certain diseases and disorders (Sivaprakasam et al., 2016; Launholt et al., 2020). On the other, acetate has more recently been cited as a significant contributor to pathologies ranging from metabolic syndrome (Perry et al., 2016) to cancer (Schug et al., 2016). One goal of this review is to help rehabilitate acetate in the eyes of biologists and highlight the positive dietary and therapeutic aspects, while also accounting for the ways in which acetate signaling and metabolism can become problematic in several common diseases. We contend that the extensive focus on the negative aspects of acetate metabolism, which is often driven by a focus on drug development, may be over emphasized in the literature. Modern diets and lifestyles predispose many people to poor health and, under those conditions, acetate and acetate precursors, such as ethanol, can contribute to morbidity. This is especially true for some cancers. When viewed from the perspective of biomass fuels or alternative energy sources, many biomolecules begin to look etiological, including acetate, glutamine, and lipids. However, acetate supplementation has also been shown to be protective in some cancers and has shown promise as an adjuvant to chemotherapy in experimental glioma (Long et al., 2015). We will attempt to reconcile some of the discrepancies in the literature, but we also caution against any sweeping generalizations because acetate metabolism and signaling are both species-specific, and exceptionally dependent upon specific metabolic and physiological stressors.

## Acetate and Health

Humans have coevolved with their gut microbiota and shifts in the human diet have had impacts on both host and microbiome (Moeller et al., 2016). The diversity of gut bacterial taxa in modern humans has become reduced relative to other primate lineages (Moeller et al., 2014, 2016). This is due in part to modern human diets, which are often poor in microbiota accessible carbohydrates (MACs). Microbiome studies indicate that modern diets in many developed countries, which tend to be low in MACs and high in meats, dairy, processed foods, and refined sugar, reduce the biodiversity of gut flora. Barone et al. (2019) found greater microbiome biodiversity in individuals who followed a modern Paleolithic diet, which approximates that observed in traditional hunter-gatherer populations, than in people who ate a standard Mediterranean diet. High-fiber diets rich in MACs are associated with distinct microbiota, including those adept at hydrolyzing, for example, fructans and galacto-oligosaccharides (So et al., 2018). Comparing the microbiota of European children to those of rural African children, De et al. (2010) found an abundance of *Prevotella* and *Xylanibacter* species in Africans that maximize energy derivation from non-digestible polysaccharides, while potentially minimizing inflammatory conditions associated with low-fiber diets. High dietary fiber intake has been associated with improved health and immune function (Daien et al., 2017). High-fiber intake has also been associated with significantly reduced human mortality (Park et al., 2011). During an average of 9 years of follow-up of a large group of people aged 50–71, high dietary fiber intake was associated with significantly lowered risk of overall death (22%) in both men (total 219,123) and women (total 168,999). Dietary fiber intake also lowered risk of death from cardiovascular, infectious, and respiratory diseases by 24–56% in men and 34–59% in women. The greatest reduction effect on mortality rates was associated with high-fiber intake from grains, as opposed to fruits, vegetables, or beans (Park et al., 2011). These findings show a clear link between high-fiber diets and improved health. The major product of bacterial fermentation of MACs in the diet is acetate, which contributes significantly to the health benefits derived from a high-fiber diet (reviewed in den Besten et al., 2013; Koh et al., 2016).

In stark contrast to studies such as these, acetate has also been linked in various ways to common health problems associated with modern, high-fat, high-calorie diets, and relatively low physical activity. These include obesity, metabolic syndrome, and non-alcoholic fatty liver disease (NAFLD), among others. Because acetate can be used for lipid synthesis, it is logical to associate it with diseases and disorders that result from a high-fat, hyper-caloric diet. Many factors have been associated with the development of metabolic syndrome and obesity, and their effects are context dependent (reviewed in Dabke et al., 2019). In a series of investigations into the potential role of acetate in metabolic syndrome and obesity, Perry et al. (2016) looked at acetate turnover and glucose-stimulated insulin secretion (GSIS) in response to hyper-caloric diets in mice. They found that increased caloric input resulted in increased acetate production and turnover. Further, they found that microbiome-generated acetate activated the parasympathetic nervous system to drive hyperinsulinemia and obesity. Thus, in mice, excessive caloric intake results in increased microbial acetate production, which in turn results in increased insulin secretion and fat deposition. When these studies were repeated in obese and lean humans using acetate infusions to increase circulating acetate levels, no effect of acetate on GSIS was observed (Petersen et al., 2019). Interestingly, acetate turnover was 30% higher in lean subjects as compared with obese subjects in the study, indicating that increased acetate throughput does not necessarily lead to obesity. These distinct findings in mice and humans highlight the often dramatic species-specific differences in acetate metabolism and signaling. Chu et al. (2019) have also noted that acetate can have opposite effects on NAFLD in humans depending on what signaling pathways are activated. We discuss acetate signaling pathways in more detail below.

It has long been known that most circulating acetate is oxidized to CO_2_ and the rest is used for lipid synthesis and acetylation reactions. Comparing radiolabeled acetate metabolism in obese and non-obese mice, it was found that the non-obese mice converted 1/3 more of the acetate to CO_2_, whereas obese mice converted twice as much acetate into fatty acids (Guggenheim and Mayer, 1952). Results such as these demonstrate that acetate metabolism is dynamic and highly responsive to changing physiological conditions. In mice fed high-fat diets, acetate suppresses hepatic fatty acid uptake, lowers serum triglycerides, reduces body fat accumulation, and improves insulin sensitivity (Weitkunat et al., 2017). In general, most animal studies and studies of human obesity have noted that increased systemic acetate leads to weight loss and improved insulin sensitivity (reviewed in Hernandez et al., 2019). Overall, the evidence favors the view that acetate from the diet or gut fermentation has positive health effects including weight reduction and improved insulin sensitivity.

### Gut Microbiota and the Liver as Two Primary Sources of Systemic Acetate

Despite many earlier researchers’ assumption that the primary source of acetate in mammals was almost exclusively due to gut fermentation, an early study comparing acetate production in various tissues of sheep and rats led to the conclusion that acetate was also produced in and released from the liver. Hepatic acetate release was found to be due to hydrolysis of acetyl-CoA, and the resultant acetate acted to redistribute an oxidizable carbon resource throughout the body (Knowles et al., 1974). As such, circulating acetate comes from three distinct sources: endogenous acetogenesis from acetyl-CoA hydrolysis, exogenous acetate from the diet (e.g., vinegar, ethanol, fermented foods, etc.), and acetogenesis from gut bacteria.

All mammals, including “foregut fermenters” such as cows and sheep, and “hindgut fermenters” such as humans and rats, exhibit various degrees of gut bacterial fermentation that generates short-chain fatty acids (Bergman, 1990). Cummings et al. (1987) examined post-mortem human samples, where acetate was detected with decreasing concentrations from the proximal colon to the distal colon, the portal vein, and then the hepatic vein, indicating that gut fermentation was the source of the acetate. They found that the hepatic vein contained 39% the level of acetate detected in the portal vein, demonstrating that the liver retained the majority of gut derived acetate, but that more than one-third was released to the circulation. These postmortem results are somewhat different from studies done in humans during surgery. Bloemen et al. (2009) showed that acetate, propionate, and butyrate were released from the gut into the hepatic portal vein and that propionate and butyrate were mostly metabolized in the liver. However, most of the acetate was not taken up and metabolized in the liver, but instead was passed to the general circulation.

Ruminants are foregut fermenters that acquire much of their nutrition through the fermentation of plant-derived polysaccharides by bacteria, which generates large quantities of the short-chain fatty acids acetate, propionate, and butyrate. Acetate is always the predominant short-chain fatty acid produced by gut bacteria, often exceeding the concentrations of all other short-chain fatty acids combined (Bergman, 1990). It is also the only short-chain fatty acid that is released in significant quantities from the gut to the general circulation in hindgut fermenters such as humans. Acetate metabolism is much more critical in ruminants than in non-ruminant mammals, and this fact had led many researchers to discount acetate metabolism in humans because the plasma levels of acetate were comparably very low (Ballard, 1972). Comparing blood levels of acetate in various mammals, Ballard (1972) reported a nearly 10-fold variation, with non-fasted goats having blood acetate levels of 1.6 mM (0.33 mM after 24 h fast), non-fasted sheep 1 mM (0.35 after 24 h fast), non-fasted pigs 0.42 mM, non-fasted rats 0.2 mM, and non-fasted humans 0.17 mM. Acetogenesis and ketogenesis have varying levels of importance in different species and in different physiological contexts. For example, in newborn pigs, plasma acetate levels are approximately 50 times higher than the levels of circulating ketone bodies (Adams and Odle, 1998). Glucose and acetate utilization for lipid synthesis in the liver of ruminants is substantially different than in non-ruminants, such as rats and humans, due to the robust production of short-chain fatty acids from microbiome-based fermentation (Hanson and Ballard, 1967).

As we noted in part 1 of this review, Pouteau et al. (2003) used intravenous ^13^C-labeled acetate infusion in healthy human adults to differentiate exogenous vs. endogenous acetate sources. They found that about two-thirds of acetate turnover involved endogenous acetogenesis, while one-third involved exogenous acetogenesis *via* bacterial metabolism in the gut. These findings indicate that, in humans, there is relatively constant output of acetate from various tissues, and that peaks in blood acetate occur on top of this steady output after meals containing fermentable fiber. The study of colonic and blood acetate levels in humans may somewhat underrepresent the evolutionary importance of this carbon source due to modern calorie dense, sugar, and starch rich diets. Many pre-agricultural and early agricultural societies likely had higher fiber content diets with extended periods of low food availability that resulted in increased reliance on gut fermentation and acetate uptake from the gut (Thorburn et al., 2014). In a modern context, it should be noted that a vegetarian diet promotes a more diverse microbiome along with the associated health benefits (reviewed in Tomova et al., 2019).

Gut fermentation of plant based polysaccharides has positive effects on cardiovascular health. Crawford et al. (2009) compared several measures of cardiac health and function in fasted, germ-free mice that lack intestinal microbiota and mice with a normal complement of gut microbes. They found that, when fasted, germ-free mice had 37% lower levels of circulating β-hydroxybutyrate, suggesting that approximately one-third of the plasma ketone bodies were derived from microbial fermentation in the gut. When fed, cecal acetate levels were 20-fold higher in mice with colonized intestines as compared with germ-free mice. Myocardial mass was significantly reduced in the germ-free mice, but this was reversed by maintenance for 30 days on a ketogenic diet. The authors note that ketone body production enhances the supply of oxidizable substrate to the heart during starvation. However, they only measured acetate levels in the cecum and did not assess plasma acetate levels, which probably would have also been elevated in the mice with colonized intestines. More recent studies in mice have confirmed a connection between high fiber diets and cardiovascular health. Marques et al. compared hypertensive (mineralcorticoid-excess) mice fed under three feeding conditions; a control diet (47.6% fiber), a high-fiber diet (72.7% fiber), and acetate supplementation (200 mM in drinking water). The high fiber diet significantly reduced systolic and diastolic blood pressures, cardiac fibrosis, and left ventricular hypertrophy. Acetate supplementation had similar effects and markedly reduced renal fibrosis. Further analysis showed that the cardiovascular effects of high fiber and acetate were accompanied by the downregulation of the transcription factor EGR1 (early growth response-1) in the heart and kidneys (Marques et al., 2017). EGR-1 is significantly involved in the cellular response to hypoxia (Yan et al., 2000). Additional evidence linking acetate availability to cardiac health comes from genome-wide association studies focused on druggable targets for coronary artery disease, which identified ACSS2 as a potential therapeutic target based on its ATP binding site (Tragante et al., 2018).

### Acetate Signaling

Metabolic regulation in response to food intake requires detailed nutrient sensing. Dysregulation of nutrient sensing occurs in a number of pathological conditions, including obesity and diabetes. Fatty acids, including acetate, act as signaling agents that are involved in regulating metabolism though distinct signaling transduction pathways. The distinct signaling functions of acetate are dependent, in part, on the source of the acetate, e.g., gut fermentation vs. intracellular deacetylation reactions that produce free acetate. We turn now to a discussion of acetate signaling involving systemic and cellular acetate availability in various diseases.

#### Diabetes

Diabetes is associated with an increase in circulating ketone bodies and acetate, but more attention has been focused on ketone bodies. In diabetic ketoacidosis, plasma ketone body concentrations can rise dramatically and the ratio of β-hydroxybutyrate to acetoacetate can shift from approximately 1:1 to as much as 10:1. Increased plasma levels of ketone bodies in diabetes are caused by increased lipolysis in adipose tissues and ketogenesis in the liver. Hormone sensitive lipase, one enzyme responsible for the increased lipolysis, is activated by both insulin deficiency and the rise in counter regulatory hormones, such as glucagon (reviewed in Laffel, 1999). Circulating free fatty acids are the principal substrates for ketogenesis and they play a major role in the increased ketogenesis in diabetes. It is noteworthy that intravenous infusion of acetate has been reported to result in a substantial (3–6-fold) drop in circulating free fatty acids in humans, and that the rebound of free fatty acid levels in blood was more rapid in subjects with normal blood insulin levels as compared with subjects with high blood insulin (Fernandes et al., 2012). The reason for the slower rebound in more insulin resistant participants is uncertain, but may be due in part to the inhibitory effect of insulin on hormone sensitive lipase activity (Meijssen et al., 2001). Transport of fatty acids into mitochondria is enhanced by glucagon-mediated reductions in cytosolic malonyl-CoA, which removes inhibition of carnitine palmitoyltransferase, the enzyme that facilitates transport of fatty acids across hepatic mitochondrial membranes. Increased fatty acid β-oxidation in mitochondria increases the levels of acetyl-CoA. However, decreased oxaloacetate concentrations caused by the decreased glycolysis in diabetes limits use of acetyl-CoA in the citric acid cycle and more acetyl-CoA is channeled toward ketogenesis, leading to large quantities of ketone bodies being released into the blood.

Glucose is the primary stimulator of insulin release from pancreatic β-cells, but other inputs contribute to the regulation of insulin secretion, including acetate. Acetate and vinegar have attracted attention in recent years as agents that can counteract several effects of diabetes and associated obesity and insulin resistance (Petsiou et al., 2014; Siddiqui et al., 2018). Ketone bodies and acetate levels are increased in diabetes due to increased fatty acid oxidation and ketogenesis caused by lack of insulin or insulin resistance. Paradoxically, administration of additional exogenous acetate in a rat model of diabetes caused marked reduction in lipid accumulation in adipose tissue, protection against accumulation of fat in the liver and improved glucose tolerance (Yamashita et al., 2007). Analysis by Northern blot revealed that transcripts of several lipogenic genes in the liver were decreased under this condition. Based on these results, Yamashita et al. hypothesized that acetate could be used to treat obesity and obesity-linked type 2 diabetes. The authors proposed that the effects of acetate involve the activation of AMPK resulting from the increased AMP/ATP ratio associated with increased acetyl-CoA formation *via* acyl-CoA short-chain synthetases (i.e., ACSS1 and ACSS2), as discussed in part 1 of this review. Yamashita et al. note that AMPK acts as a key metabolic switch that regulates enzymes involved in lipid metabolism and point to studies showing that carbohydrate-responsive element binding protein (ChREBP) is also phosphorylated by AMPK. ChREBP is a transcription factor for enzymes involved in lipogenesis, and AMPK mediated phosphorylation of ChREBP inhibits activity, resulting in the decreased transcription of lipogenic enzyme genes (reviewed in Yamashita et al., 2007). Acetate administration was also shown to reduce the size of intracellular lipid droplets in the same rat model of diabetes, and the authors propose that acetate levels act, in general, to counter the effects of reduced insulin and associated fat accumulation (Yamashita et al., 2009). In a review of studies on the effects of vinegar in diabetic and non-diabetic humans, it was concluded that vinegar lowers post-prandial glucose levels and increases insulin sensitivity in non-diabetics, but did not have the same positive effects in diabetic patients (Lim et al., 2016). This contrast highlights the context-specific beneficial effects of acetate on human health, where acetate metabolism or signaling can have distinct effects depending on whether an individual is healthy or has an underlying disease.

#### Free Fatty Acid Receptors and Diabetes

For many years the effects of acetate on physiology were thought to be mediated almost exclusively through acetyl-CoA formation. While it is true that acetyl-CoA formation from acetate can generate changes in physiology in all cell types, the discovery of a family of short-chain fatty acid receptors that signal through G proteins revealed that fatty acids, including acetate, could have direct transduction effects in cells that express one or more of these receptors. In a classic example of scientific serendipity in the search for the endogenous ligands of two so-called “orphan G-protein coupled receptors,” GPR41 and GPR43, Brown et al. (2003) applied acetate salts of various potential ligands to screen for possible activators. All of the acetate salts activated the receptors and, after further investigation, they found that sodium acetate was a potent ligand. Similar results were reported in the same year by two other laboratories demonstrating that all short-chain fatty acids activated these receptors (Le et al., 2003; Nilsson et al., 2003). In particular, GPR43 exhibited an EC_50_ (half maximal effective concentration) of 52 ± 10 μM for acetate as the activating ligand, indicating that the receptor would be activated by physiological levels of acetate.

Additional research into these receptors has provided crucial information on their links to acetate signaling and control of various aspects of metabolism, including links to diabetes and insulin release (reviewed in Layden et al., 2013; Priyadarshini et al., 2018; Santos et al., 2019). GPR43 and GPR41 are both short-chain fatty acid receptors and have therefore been redesignated as free fatty acid receptors 2 and 3, respectively (Stoddart et al., 2008). Inconsistencies in the use of acronyms for these receptors are common in the literature. The official gene acronyms (HUGO Gene Nomenclature Committee) are *FFAR2* and *FFAR3*. However, the receptors are often designated as FFA2 and FFA3 (Stoddart et al., 2008; Priyadarshini et al., 2018). We will use the official gene acronyms for both the genes and proteins; italics for the genes and non-italics for the receptors.

There are four known free fatty acid receptors coupled to G-protein signaling systems; FFAR1-FFAR4, and all four have been proposed as therapeutic targets for the treatment of diabetes (reviewed in Offermanns, 2014; Watterson et al., 2014; Kimura et al., 2020). FFAR1 (aka GPR40) responds to medium- and long-chain fatty acids (C6–C22), whereas FFAR4 (aka GPR120) is activated by long-chain fatty acids (C14–C22) including omega-3 and omega-6 fatty acids (Offermanns, 2014). FFAR2 and FFAR3 respond to some degree to all the short-chain fatty acids (C2–C6), but they have somewhat different affinities based on chain length. FFAR2 is most responsive to acetate and propionate, whereas FFAR3 favors propionate, butyrate, and pentanoate (Brown et al., 2003; Le et al., 2003; Nilsson et al., 2003). However, the mouse and human receptors have quite different ligand affinities. Acetate is approximately 20-fold more potent at human FFAR2 than at human FFAR3, but has similar potencies at the two mouse receptors. Also, propionate is more potent at mouse FFAR3 than FFAR2, but shows similar potency for the two human receptors (Hudson et al., 2013). This emphasizes that functional extrapolation from mice to humans must be done cautiously taking into consideration that systemic acetate levels and receptor sensitivities are species specific (Priyadarshini et al., 2015).

In the intestines, FFAR2 and FFAR3 have mostly distinct expression patterns, with FFAR2 being present in some enteroendocrine cells and many leukocytes in the lamina propria, whereas FFAR3 is present in enteroendocrine cells and the neuronal cells of the submucosal and myenteric ganglia (Tolhurst et al., 2012; Nohr et al., 2013). Both receptors are localized in pancreatic β-cells and have been linked to glucose-stimulated insulin release in mice (McNelis et al., 2015; Tang et al., 2015). However, results indicate that acetate can either inhibit or enhance insulin release in response to glucose. There are differences between the activity of the mouse and human receptor systems (reviewed in Priyadarshini et al., 2016, 2018) and the inconsistencies in reported results are likely due to at least two different mechanisms. First, responses depend on which G-protein the receptor signals through (Gα_i/o_ vs. Gα_q11_). Further, substantial differences have been found when comparing responsiveness of mouse and human FFAR2 (Priyadarshini et al., 2015). Mouse FFAR2 is not constitutively active, whereas human FFAR2 is, and therefore the human receptor does not respond to ligands to the same degree as the mouse receptor (Hudson et al., 2012). Second, the conflicting findings on acetate and insulin secretion may be due to the fact that FFAR2 and FFAR3 can form a heterodimer with different functional characteristics. For example, the FFAR2-FFAR3 heterodimer lost the ability to inhibit cAMP production, but gained the ability to induce p38 phosphorylation, shifting the functional outcome of acetate binding to the receptor (Ang et al., 2018).

### Two Modes of Acetate Signaling

**Figure 1:**
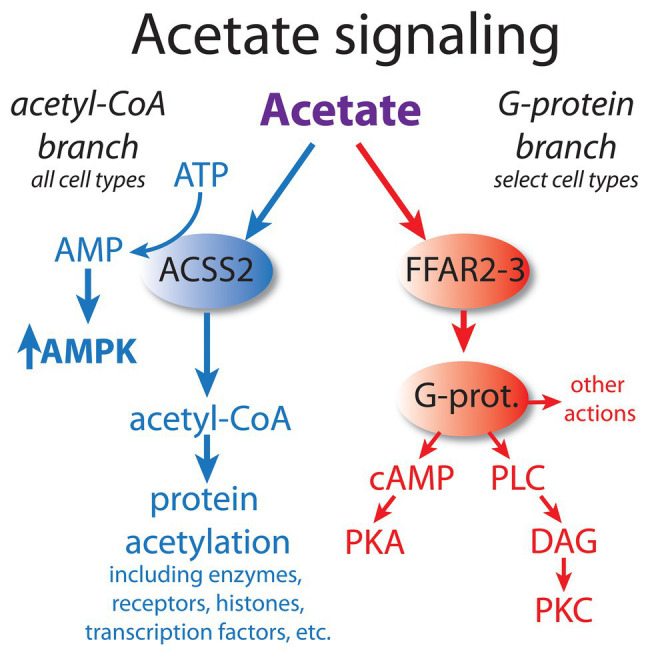
Acetate acts *via* at least two major signaling pathways. Based on their mode of action, we propose that they can be conceptualized as the acetyl-CoA signaling branch and the G-protein signaling branch. The acetyl-CoA branch involves regulation of protein function and gene transcription *via* acetylation/deacetylation cycles, as well as through AMPK kinase cascades. This signaling system is operative in all cell types, but the responses are cell type-specific. In contrast, the G-protein branch signals through cyclic AMP (cAMP) and phospholipase C (PLC) and is restricted to certain cell types. The acetyl-CoA branch is linked to the integrated stress response (ISR) associated with oxygen and nutrient deprivation, whereas the G-protein branch involves metabolic regulation based on fatty acid levels. Dysregulation of both pathways occurs in cancer. AMPK, adenosine monophosphate kinase; cAMP, cyclic AMP; DAG, diacylglycerol; FFAR2 and 3, free fatty acid receptors; G-prot., G proteins; PKA, protein kinase A; PKC, protein kinase C; and PLC, phospholipase C.

At least two major and distinct acetate signaling pathways can be distinguished. As discussed above, one major signaling pathway acts through free fatty acid receptors, particularly FFAR2. This pathway signals through G-proteins and subsequently cAMP or phospholipase C, which then target protein kinase A (PKA) and protein kinase C (PKC) activity, respectively. The other major acetate signaling pathway acts through the formation of acetyl-CoA, which can then affect many cellular functions *via* the targeted acetylation of enzymes, receptors, histones, and transcription factors (see part 1 of this review for details). These two major acetate signaling systems can be conceptualized as the acetyl-CoA and G-protein pathways (Figure 1). The acetyl-CoA signaling branch primarily operates through ACSS2, which generates acetyl-CoA in the cytoplasm and nucleus of most, or perhaps all cell types. The mitochondrial form of the enzyme, ACSS1, is more involved in energy derivation *via* the tricarboxylic acid (TCA) cycle, but may also provide acetyl-CoA for intramitochondrial acetylation reactions. The acetyl-CoA signaling branch is ubiquitous, and operates in virtually all cell types, whereas the G-protein branch is more restricted in its distribution in various tissues. For example, the G-protein acetate signaling pathway is particularly active in cells of the immune system, where it regulates inflammatory responses. This has been found to be the case in T regulatory cells in the colon, where FFAR2 activity protects against colitis (Smith et al., 2013). The enhanced expression of the G-protein acetate signaling pathway in the immune system indicates that acetate can regulate certain inflammatory responses.

### Acetate Signaling and Inflammation

Microbiome-generated short-chain fatty acids have been implicated in modulating immune responses, linking diet to the regulation of inflammation. Acetate modulates inflammation through both the G-protein and acetyl-CoA signaling pathways involving FFAR and ACSS2, respectively. Using FFAR2-deficient mice (*Gpr43* ^−/−^), Maslowski et al. (2009) demonstrated that acetate derived from gut microbiota reduced inflammation in mouse models of arthritis, colitis, and asthma. In all of these models, the FFAR2-deficient mice exhibited increased or prolonged inflammatory responses. These investigators further showed that experimental colitis was significantly exacerbated in germ-free mice, and that acetate supplementation in the germ-free mice reduced colonic inflammatory responses. FFAR2 is expressed strongly in leukocytes (Nilsson et al., 2003) and is activated by acetate and propionate to initiate calcium mobilization in peripheral immune cells such as human polymorphonuclear leukocytes (Nakao et al., 1992). Acetate also acts to reduce inflammasome activation *via* FFAR2 (Xu et al., 2019). As such, FFAR 1–4 are all potential therapeutic targets in inflammatory bowel diseases (Bartoszek et al., 2020). In contrast to the anti-inflammatory actions of acetate in some model systems, acetic acid infusion directly into the colon strongly induces inflammation, and has long been used as an experimental model for ulcerative colitis (Tahan et al., 2011; Elshazly et al., 2020). Such contradictory results indicate the context-specific nature of acetate signaling.

Most of the research into the anti-inflammatory effects of acetate has focused on signaling through free fatty acid receptors. However, there is evidence that acetate can also regulate inflammatory responses through the acetyl-CoA signaling branch *via* the action of ACSS2. Transcriptome analyses into the effects the so-called “cafeteria” (CAF) high-fat, high-calorie diet on gene expression in monocytes from two different rat strains found that ACSS2 is at the hub of a nutrient-sensing system that impacts on inflammatory responses (Martinez-Micaelo et al., 2016a,b). Monocytes from the Lewis rat strain only showed modest transcriptional changes in response to the CAF diet, whereas monocytes from the Kyoto Wistar rat strain exhibited robust transcriptional changes. Examination of the expression level of ACSS2 from monocytes of the two rat strains maintained under the CAF diet showed an inverse correlation with blood glucose, triglyceride, non-esterified fatty acid, and leptin levels. ACSS2 expression was also inversely correlated with total fat weight and body weight. As such, high ACSS2 expression was associated with reduced energy stores. Enrichment analysis of the differentially expressed genes between CAF-fed Kyoto Wistar and Lewis rats showed that the most overrepresented gene ontology (GO) categories included immune response, response to cytokines, response to stress, and inflammatory response (Martinez-Micaelo et al., 2016b). Another study done in murine white blood cells showed that low ACSS2 expression was correlated with a higher degree of glucose intolerance (Wopereis et al., 2012). These findings further highlight the links between ACSS2 nutrient sensing and inflammatory responses.

### Acetate From Ethanol

Ingested ethanol is converted to acetate in two enzymatic steps (Zakhari, 2006) catalyzed by alcohol dehydrogenase (EC 1.1.1.1) and aldehyde dehydrogenase 2 (EC 1.2.1.3). Both reactions involve the reduction of NAD+ to NADH. Ethanol is also produced, usually at very low levels, by fermentation in the gut and is therefore a minor contributor to acetate formation in all mammals. Common gut bacteria including *Escherichia coli* produce ethanol from glucose (Dawes and Foster, 1956). An examination of ethanol production from common gut microbes *in vitro* showed that a number of species produced significant quantities of ethanol (Elshaghabee et al., 2016). In healthy humans, the production of ethanol by the intestinal microbiota is very low, but can become significant in some pathological cases such as diabetes mellitus (Simic et al., 2012). Increased ethanol production from abnormal gut microbial action has been implicated as one etiological factor in non-alcoholic fatty liver disease (Smith, 2012; Zhu et al., 2013; Yuan et al., 2019). It is likely that all microbiome-generated ethanol is processed to acetate on first-pass metabolism in the liver, but in healthy humans this contribution is probably negligible relative to the total acetate production in the liver.

Ethanol consumption leads to a proportional increase in circulating acetate (Mascord et al., 1992). Using liver perfusion experiments in rats fasted for 24 h it was shown that as the level of ethanol taken up from the perfusate increased from 2.2 to 10.4 μmoles/min/liver, and the acetate production increased from 1.7 to 9.1 μmoles/min/liver. This demonstrates rapid and efficient conversion of ingested ethanol to acetate in the liver (Yamashita et al., 2001). The substantial increase in systemic acetate associated with alcohol consumption has been shown to have significant effects on protein acetylation. Alcohol metabolism to acetate has been shown to increase histone H3 acetylation at the lysine-9 position (H3K9ac) in a dose-dependent manner in the nuclei of rat hepatocytes (Park et al., 2003). Blocking the activity of either alcohol dehydrogenase or aldehyde dehydrogenase greatly reduced H3K9ac in response to alcohol treatment. In these studies, other H3 acetylation sites were unaltered in the presence of ethanol. Treatment of a human monocyte cell line with acetate (1 mM) or ethanol (86 mM) led to increased histone acetylation (Kendrick et al., 2010). Because histone acetylation is associated with chromatin remodeling during gene activation, ethanol can exert multiple effects on the epigenetic regulation of transcription (reviewed in Zakhari, 2013). In addition to nuclear acetylation, cytoskeletal and mitochondrial proteins are also hyper-acetylated in response to ethanol treatment (Shepard et al., 2010). Due to its role in acetate metabolism and signaling, it is not entirely surprising that ACSS2 has been implicated in alcoholism. Ribeiro et al. (2017) gave mice access to water and water with alcohol and divided the mice into behavioral groups they named “compulsive-like drinker,” “ethanol-preferring drinker,” and “ethanol-non-preferring drinker.” They found that *ACSS2* mRNA levels in the amygdala were significantly elevated only in the compulsive-like drinker group. Further, examination of the *ACSS2* gene in human volunteers suggested that the single nucleotide polymorphism, rs6088638, was common in alcoholics.

More recently, Mews et al. (2019) administered mass-labeled acetate (acetate-d3) and alcohol (ethanol-d6) to show that both resulted in extensive histone acetylation in the hippocampus and prefrontal cortex. As with previous studies, increased H3K9ac was observed in response to both acetate and ethanol treatment, and they also observed H3K27 acetylation. It is noteworthy that the labeling was rapidly removed from the histones indicating the dynamic nature of this regulatory response. Using a neuronal cell culture model, the authors demonstrated that acetate supplementation induced transcriptional regulation of various genes, which was blocked by the presence of an ACSS2 inhibitor. ChIP-seq analysis showed ACSS2 bound to gene promoter regions for neuronal genes associated with learning and memory (Mews et al., 2017). Also in neuronal cell culture, ACSS2 expression in differentiating neurons increased in the nuclei and localized to upregulated neuronal genes near sites of elevated histone acetylation. Under these conditions, inhibiting ACSS2 lowered nuclear acetyl-CoA levels, histone acetylation, and expression of specific neuronal genes. In adult mice, reduction of hippocampal ACSS2 expression impaired long-term spatial memory through effects on histone acetylation (Mews et al., 2017). These examples highlight the fact that the role of ACSS2 appears to be cell-type specific. Different cell types express different repertoires of accessible histone acetylation sites and operational transcription factors, only some of which are regulated by ACSS2 and increased acetate levels. This may explain some of the discrepancies in the literature on the effects of acetate and ethanol.

### Dietary Acetate Supplementation With Glyceryl Triacetate

Acetate administration can be used to study the effects of high blood and tissue acetate levels. One of the most effective methods identified for substantially increasing acetate levels is to deliver it in the form of the acetate triester of glycerol (glyceryl triacetate or GTA), also known as Triacetin®. GTA is a synthetic short-chain triglyceride (two carbon chain length), which is thought to be metabolized by the same lipases and esterases responsible for cleaving other fatty acids from triglycerides. Upon complete hydrolysis, three acetate molecules are released per GTA molecule, along with one molecule of glycerol. As an intact molecule, GTA is relatively small and hydrophobic and therefore may cross cell membranes to some extent without the aid of transporters or carriers, but this has not been investigated directly.

GTA was investigated by NASA in the 1960s as a potential nutrient for prolonged spaceflight. NASA scientists wanted to determine what proportion of the diet could be composed of artificial nutrients that were easy to synthesize chemically in space. They fed several compounds to rats including GTA and found that the diet could be composed of up to 20% GTA without adverse effects (Shapira et al., 1969). Higher levels of GTA in the diet led to weight loss. Studies continued into the 1990s on the possible use of GTA as a parenteral nutrient for use in patients unable to take food by mouth (Bailey et al., 1991, 1992). Subsequently, it was noted that including GTA as a significant proportion of the diet did not alter the caloric intake, but did result in weight loss and reduced fat deposition in rats (Lynch and Bailey, 1995), suggesting that increased GTA or acetate levels led to increased lipolysis and β-oxidation and decreased fat synthesis. As noted above, acetate reduces insulin secretion and this may partly underlie acetate-induced weight loss.

During investigations into the use of various ketone body precursors as a method of improving outcomes after brain injury, Robertson et al. (1992) found that inducing ketogenesis *via* the diet was beneficial. They tested compounds including GTA, 1,3-butanediol, tributyrin, and medium and long-chain triglycerides in the diet of rats subjected to experimental cerebral ischemia and found that 1,3-butanediol and tributyrin plus GTA were the most effective at reducing the size of the cerebral infarct. In another study, high dose GTA (7.5 gm/kg) given to rats immediately after experimental brain injury increased the levels of ATP and NAA in the brain and improved motor performance, indicating that GTA has neuroprotective effects (Arun et al., 2010a).

GTA has also been tested as a treatment for the fatal genetic disease Canavan disease, which is caused by mutations in the gene for the NAA catabolizing enzyme aspartoacylase (ASPA), one of the aminoacylase enzymes that act to remove acetate from N^α^-acetylated amino acids (see part 1 of this review for more details on NAA and ASPA). Lack of ASPA enzyme activity in the brain during development causes a buildup of NAA, reduced myelination, and eventually brain vacuolation and death (Hagenfeldt et al., 1987; Matalon et al., 1988). In a rat model of Canavan disease, GTA administration improved motor function and increased myelin-associated lipids, but vacuole formation in the brain was only modestly reduced (Arun et al., 2010b). The authors hypothesized that the GTA-derived acetate substituted for the lack of NAA-derived acetate, and that this partially rescued oligodendrocytes and improved myelin lipid synthesis. Previous studies had shown that histone H3 acetylation was increased in Canavan disease (Kumar et al., 2009) leading Arun et al. to suggest that disrupted histone acetylation could be involved in the pathogenesis of Canavan disease, and that GTA may have acted to partially rectify the dysfunction (Arun et al., 2010b). Subsequently, it was reported in the rat model of Canavan disease that H3K9ac was substantially increased in the nuclei of oligodendrocytes and that this hyper-acetylation was paradoxically reduced to near normal levels with GTA treatment (Moffett et al., 2014). These findings suggest that GTA-derived acetate is not acting simply by mass action to increase histone acetylation levels, but is acting to regulate acetylation of specific histone residues, most likely through ACSS2.

Similar to the case with ethanol-derived acetate, GTA treatment was found to increase histone acetylation levels in the rat brain at the H3K9 acetylation site (Soliman and Rosenberger, 2011). Further, acetylation of histone H4 was observed in the rat brain at lysine sites 8 and 16. Importantly, increased histone acetylation was not observed in the liver of the same rats, most likely because acetate is not utilized extensively in the liver. Acetylation of the H3K9 position is strongly associated with increased acetate levels and it is likely that this will become a recurrent theme in the study of the numerous roles that acetate plays in signaling and gene regulation through targeted transcription factor activation. H3K9 and H3K14 acetylation correlate with the regulatory elements of many genes (Karmodiya et al., 2012).

In other studies, GTA has been found to have additional effects in the central nervous system (CNS), including anti-inflammatory actions. Lipopolysaccharide-induced neuroinflammation was reduced by high dose GTA treatment (Reisenauer et al., 2011), which also reduced microglial activation and IL1-B levels in experimental Lyme disease (Brissette et al., 2012). GTA administration also decreased brain histone deacetylase (HDAC) expression and modulated histone acetylation levels (Soliman and Rosenberger, 2011; Soliman et al., 2012). Further, GTA was found to increase brain adenosine A2A receptor expression levels (Smith et al., 2014). Additional studies have indicated that GTA has specific effects on brain energy systems. For example, GTA administration increased phosphocreatine levels and reduced AMP levels in the brain (Bhatt et al., 2013). We conclude that GTA and acetate have neuroprotective effects in the brain by improving specific aspects of energy status and by reducing neuroinflammation. Similar to the use of GTA as an acetate delivery vehicle, esters of glycerol and ketone bodies have been investigated as a ketogenic treatment for various diseases and disorders, including epilepsy (reiewed in Hashim and Vanitallie, 2014).

Considering the rapid system-wide acetylation of proteins in response to acetate treatment, and the subsequent rapid deacetylation that follows, it is tempting to propose that diet, in concert with the gut microbiome, act to load histone and non-histone proteins with associated post-translational modifications (PTMs) to fine tune gene transcription and protein function accordingly. Different PTMs such as acetylation, propionylation, butyrylation, crotonylation, and succinylation, can have distinct effects on gene transcription (Barnes et al., 2019). For example, acetate reliably leads to H3K9ac, which is associated with transcriptional activation for specific genes associated with response to stress or nutritional deprivation, as well as memory formation in the brain. This site is very sensitive to systemic acetate levels and therefore links diet, liver metabolism, and gut fermentation to selective transcriptional activity (Mews et al., 2019). The various short-chain fatty acid PTMs may be biasing the system epigenetically to adapt to the nutritional and health state of the organism. When considered in the context of acetate effects on learning and memory, it is also tempting to speculate that this acts to help imprint the CNS with critical information on available food sources. The effect would lead to enhanced memory consolidation about the seasonal locality of food sources to be found in the environment. This could be especially important during times of low food availability, where the increase in acetate levels after a meal could facilitate memory formation about the food source.

## Acetate in Cancer

**Figure 2:**
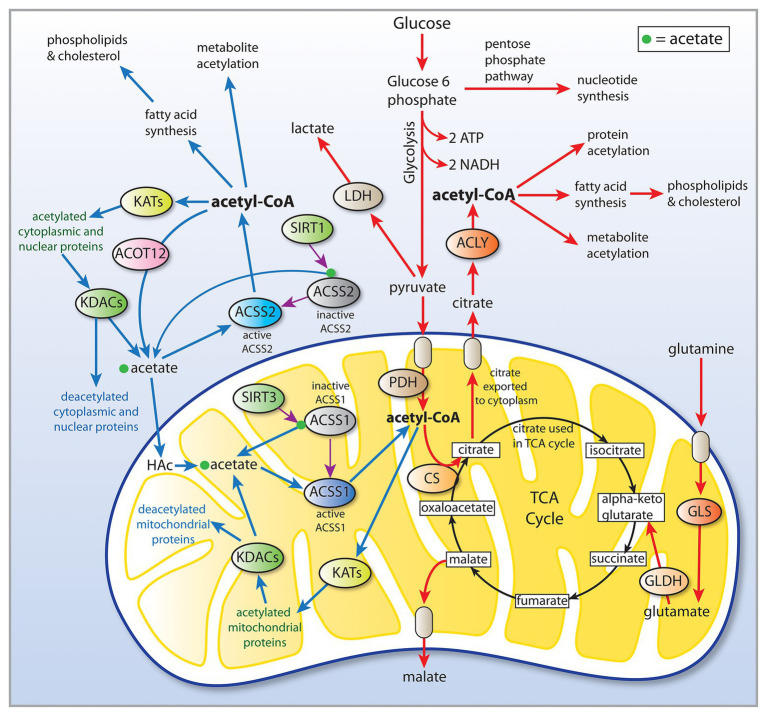
Central carbon metabolism and links to ACSS function. Inputs to the TCA cycle come from numerous sources, including glucose, beta-oxidation of fatty acids, and metabolism of amino acids. Glucose is converted to pyruvate *via* glycolysis and the pyruvate then enters the TCA cycle after conversion to acetyl-CoA by the pyruvate dehydrogenase complex (PDH). The condensation of oxaloacetate with acetyl-CoA by citrate synthase (CS) generates citrate, which can either be oxidized to CO_2_ in the cycle for energy derivation or the citrate can be exported to the cytoplasm. In the cytoplasm, citrate is reconverted to acetyl-CoA by ATP citrate lyase (ACLY). This generates the bulk of the cytoplasmic and nuclear pools of acetyl-CoA. The ACSS enzymes, in contrast, generate acetyl-CoA directly from free acetate in all cellular compartments. This provides cells with distinct routes to acetyl-CoA formation that can be regulated differentially. ACLY, ATP citrate lyase; ACOT12, acyl-CoA thioesterase 12; ACSS1, acyl-CoA short chain synthetase family member 1; ACSS2, acyl-CoA short chain synthetase family member 2; CS, citrate synthase; GLDH, glutamate dehydrogenase; GLS, glutaminase; KATs, lysine acetyltransferases; KDACs, lysine deacetylases; LDH, lactate dehydrogenase; SIRT1, sirtuin 1; and SIRT2, sirtuin 2.

Cancer involves genetic, signaling, and epigenetic changes in cells that lead to the alteration of basic metabolism, including how cells derive energy from various carbon sources. Cancer cells often exhibit increased aerobic glycolysis and reduced oxidative phosphorylation, which has been known as the Warburg effect (Bayley and Devilee, 2012). Warburg reported in 1927 that tumors used approximately two-thirds of blood glucose for fermentation (glycolysis) and one-third for oxidative respiration (Warburg et al., 1927). These findings were originally interpreted to indicate that mitochondria had become defective and therefore cancer cells relied on increased glycolysis for ATP generation. Other metabolic alterations include increased glutaminolysis (DeBerardinis et al., 2007) and increased lactate production. However, these metabolic alterations are typical of non-transformed, rapidly dividing cells, which tend to forego ATP production through oxidative phosphorylation in favor of rapid turnover of glucose in combination with increased pentose-phosphate shunt throughput, leading to lactate production. How these alterations are faithfully manifested in cancer cells, and how they are maintained through repeated cell cycles is the focus of intensive research into the disrupted epigenetic regulation of metabolism and cell cycle control. Enzyme activity, transcription factor activation, and protein interactions are regulated by acetylation and deacetylation of specific lysine sites on the surface of proteins. The control of intermediary metabolism in mitochondria is regulated in part by acetylation. Similarly, acetylation of histone and non-histone proteins in the nucleus is a key factor in epigenetic regulation of gene transcription through selective chromatin remodeling and transcription factor activity. Protein acetyltransferases (KATs) and deacetylases (KDACs) act as key regulators of these processes. Sirtuins, which are NAD+-dependent protein deacetylases, also regulate cell behavior *via* selective deacetylation (see part 1 of this review for details), including acting to deacetylate ACSS1 and ACSS2 (Figure 2).

Acetate and ketone bodies are readily utilizable carbon sources for cell growth, and therefore are theoretically capable of facilitating cancer cell proliferation. However, some cancer cells appear less capable of utilizing ketone bodies for energy derivation than normal cells (Maurer et al., 2011; Meidenbauer et al., 2015). Therefore, the use of a ketogenic diet has emerged as an adjuvant approach for the management of cancers with a very poor prognosis, such as glioma (Seyfried and Mukherjee, 2005; Seyfried et al., 2011, 2014). The reduced capacity to utilize ketone bodies in cancer cells is thought to be due to their altered mitochondrial function. In addition to ketone body oxidation, acetate oxidation also occurs in mitochondria *via* ACSS1 and would therefore be reduced if mitochondrial respiration was impaired.

Acetate plays several roles in cancer including oxidation in mitochondria for energy derivation, lipid synthesis in the cytoplasm, and protein acetylation in all subcellular compartments, including histone and transcription factor acetylation in the nucleus. Because the latter role is purely regulatory, rather than being tied to biomass accumulation, acetate can have complex and seemingly contradictory effects on tumor cells. Both ACSS1 and ACSS2 have been implicated in acetate utilization by cancer cells. For example, among several hepatocellular carcinoma cell lines, it was found that some preferentially took up and metabolized acetate, others preferred glucose, and some utilized both substrates (Yun et al., 2009). In SNU449 carcinoma cells, which strongly preferred acetate over glucose, knockdown of *ACSS1* and *ACSS2* reduced acetate uptake and cell viability. Importantly, knockdown of *ACSS2* had the most significant effect on cell viability resulting in over 90% cell death compared with controls. Even in the case of lipogenesis, acetate acts as an epigenetic regulator of lipid synthesis *via* ACSS2 under hypoxic conditions in cancer cells derived from hepatocytes (Gao et al., 2016). Gao et al. showed that acetate induced a greater increase in ACSS2 mRNA expression than any other gene *in vitro* under hypoxic conditions. Further, acetate acted to promote increased expression of lipogenic enzymes by hyper-acetylation of histones H3K9, H3K27, and H3K56. In glucose-deprived T cells, acetate facilitates histone acetylation, increases IFN-γ production, and promotes tumor clearance (Qiu et al., 2019). In general, evidence is mounting that the role of ACSS2 shifts during oxygen and nutrient deprivation from lipogenesis to metabolic and signaling regulation *via* transcription factor complexes.

Under normoxic conditions in cancer cells, the major carbon sources for biomass accumulation, including lipid synthesis, are derived through glycolysis coupled with glutaminolysis. In contrast, under hypoxic conditions the reliance on glucose and glutamine as carbon sources in cancer cells is reduced, with acetate being cited as a substitute carbon input for lipid synthesis (Kamphorst et al., 2014). Major sources of acetyl-CoA in cancer cells in rank order appear to be glucose, amino acids including glutamine and glutamate, and acetate (Kamphorst et al., 2014). Schug et al. (2015) showed that under hypoxia and low serum conditions the shift toward acetate utilization in cancer cells was associated with the labeling of fatty acids and lipids and suggested that ACSS2 was needed for fatty acid synthesis when cancer cells proliferate under metabolically stressed conditions. Additional stressors include nutrient deprivation and extracellular acidification due to factors such as aerobic glycolysis, excess lactate production, and elevated CO_2_ (Damaghi et al., 2013). Recently it has been found that extracellular acidification activates SREBP2, which, in turn, increases expression of ACSS2, and this provided a growth advantage to cancer cells exposed to acidic pH (Kondo et al., 2017).

Ketone bodies are not utilized extensively in most cancer cells as long as glucose is available. It is now thought that the mitochondria in cancer cells are not defective as Warburg proposed, but are locked in a particular metabolic state associated with rapid cellular proliferation linked to high glucose and glutamine availability. When cells rely primarily on glucose for aerobic glycolysis, there is no need to activate salvage systems associated with nutrient deprivation, including both ketone body and acetate utilization. But cancer cells, like all cells, may be able to adapt to low carbohydrate, low protein and high fat conditions over time. Ketogenic diets are not effective in reducing cancer growth unless they are coupled with calorie restriction (Seyfried et al., 2015). With minimally sufficient caloric intake and a significant shift to fat content, cancer cells have limited access to glucose and amino acids including glutamine. A recent small scale study showed that combining a ketogenic diet with chemotherapy in patients with advanced local or metastatic breast cancer improved survival rates (Khodabakhshi et al., 2019).

Due to its role in stress-response signaling and regulation, acetate might be expected to have anti-cancer effects in addition to pro-cancerous effects, depending on the cell type of origin and the tumor microenvironment. For example, acetate has been shown to have anti-cancer effects in colon cancer, adrenocarcinoma, and glioma cell lines, although the molecular mechanisms underlying these effects remain poorly understood (Long et al., 2013b, 2015; Marques et al., 2013; Xia et al., 2016). High concentrations of acetate have also been shown to be cytotoxic to thymic tumor cells *in vitro*, whereas the same concentration of acetate (12.5 mM) was not toxic to normal thymocytes (Pandey et al., 2018a). Super-physiological levels of acetate are required for this effect, so while not relevant in normal physiology, acetate supplementation could provide an adjunct therapy to the treatment of certain cancers (Tsen et al., 2014). Possible mechanisms for acetate cytotoxicity to certain cancer cell types will likely turn out to be cell type specific. These could involve acetate mediated increase in lysosomal and mitochondrial permeabilization (Marques et al., 2013). Also, acetate is known to induce apoptosis in some cancer cells in a caspase-dependent manner (Xia et al., 2016). In many cases, the cell-specific effects may be through acetate-based epigenetic mechanisms, regulating gene transcription. Because ACSS2 activity is inhibited by acetylation (Hallows et al., 2006), it would be expected that very high levels of acetate could act to limit activity through this pathway, thus impacting lipid synthesis and the acetylation of histones and transcription factors.

In yeast, both acetate and acetyl-CoA promote growth by specific acetylation of histones associated with growth-related genes (Cai et al., 2011). The mechanisms by which specific lysine residues are acetylated by various acetyltransferase enzymes have not been well studied. CREB binding protein (CBP) and p300 are homologous lysine acetyltransferases that acetylate histone proteins. To investigate how acetyltransferases such as CBP and p300 could act selectively on specific histone lysine sites, Henry et al. (2015) used an inhibitor of p300 designated C646. C646 competes with acetyl-CoA for binding to the enzyme and by varying concentrations of C646, they were able to demonstrate that p300 has distinct conformations that alter its substrate specificity. They showed that acetyl-CoA levels shift the substrate specificity of p300, and this alters which histone lysine sites get acetylated. Because ACSS2 can increase the level of acetyl-CoA locally in the nucleus, where protein deacetylation is robust, ACSS2 activity shifts the set of histone sites that become acetylated based on local substrate availability. As such, local acetate in the nucleus will drive shifts in substrate preference for histone acetyltransferases. This provides additional flexibility in how ACSS2 links nutrient levels to transcription. Further, the set of transcription factors that are acetylated by ACSS2 will no doubt shift based on various physiological stressors. It is likely that the shifts in both histone and transcription factor acetylation are coordinated to open the correct chromatin sites for the associated transcription factors to gain access to appropriate promoter sites.

### ACSS Enzymes in Cancer

As noted in part 1 of this review, there are three ACSS enzymes, ACSS1, ACSS2, and ACSS3. All of them have been associated with cancer progression. The mitochondrial forms, ACSS1 and ACSS3, most likely play a significant role in energy derivation *via* oxidation of acetate and propionate, respectively, but may be less involved in regulatory functions in cancer cells than ACSS2. One possibility that has not been investigated to our knowledge is that ACSS1 facilitates specific acetylation reactions in the mitochondrial matrix. In such a scenario, ACSS1 would bind to specific protein complexes in the matrix and directly provide acetyl-CoA for targeted acetylation reactions, which could alter the TCA cycle and central metabolism.

One study of human hepatocellular carcinomas showed that tumor subtypes could be stratified based on acetate metabolism (Bjornson et al., 2015). Interestingly, increased expression of ACSS1 (the mitochondrial form involved in acetate oxidation) was associated with the tumor subtype with the lowest patient survival rate. In a subsequent study, the three different ACSS enzymes were each identified as one of the network hub genes in the three distinct hepatocellular tumor subtypes, iHCC1–iHCC3 (Bidkhori et al., 2018). ACSS1 was associated with iHCC3, which had the lowest survival rate, whereas ACSS2 was associated with the moderate survival rate cancers (iHCC2) and ACSS3 was associated with the subtype having the longest patient survival rate (iHCC1). As such, the two mitochondrial forms were associated with the lowest and highest survival groups, suggesting distinct roles for these similarly localized isoforms. This may be due to their different substrate preferences (acetate for ACSS1 vs. propionate for ACSS3). Chang et al. (2018) used bioinformatic analyses to show that ACSS3 expression is associated with disease progression in gastric cancer patients. They also showed that growth of human gastric adenocarcinoma cells in culture could be blocked by a combination of nutrient deprivation and *ACSS3* knockdown. The role of ACSS3 in the generation of lipogenic acetyl-CoA has been examined in bladder urothelial carcinoma cells (BLCA) under metabolic stress. ACSS2 and ACSS3 protein expression levels were increased *in vitro* by reduced serum and reduced oxygen, whereas the expression of ACSS1 was not changed by either one (Zhang et al., 2020). Knockdown of *ACSS3* in these cells resulted in reduced labeling of fatty acids from radiolabeled acetate, suggesting that ACSS3 provided acetyl-CoA for lipid synthesis. Further, histone acetylation was reduced by *ACSS3* knockdown. Both of these studies (Chang et al., 2018; Zhang et al., 2020) implicated mitochondrial ACSS3 with the generation of acetyl-CoA in the cytoplasm and nucleus. This could be accomplished if the acetyl-CoA generated by ACSS3 was utilized by citrate synthase, followed by export of citrate to the cytoplasm and then the nucleus. Because this route of acetyl-CoA formation would require the action of ATP citrate lyase (ACLY), rather than acetyl-CoA formation through ACSS2, future studies on ACSS3 and lipogenesis should include ACLY inhibitors to confirm that this pathway does not involve ACSS2. It is likely that in some cancers this route of acetate utilization is important. However, these results are in conflict with enzyme assays that show ACSS3 preferentially utilizes propionate as substrate (Yoshimura et al., 2016). These discrepancies will have to be reconciled by future studies that demonstrate whether ACSS3 can indeed utilize acetate at concentrations and conditions found in the mitochondrial matrix, and that ACSS1 is not the preferred route in some cancers.

**Figure 3:**
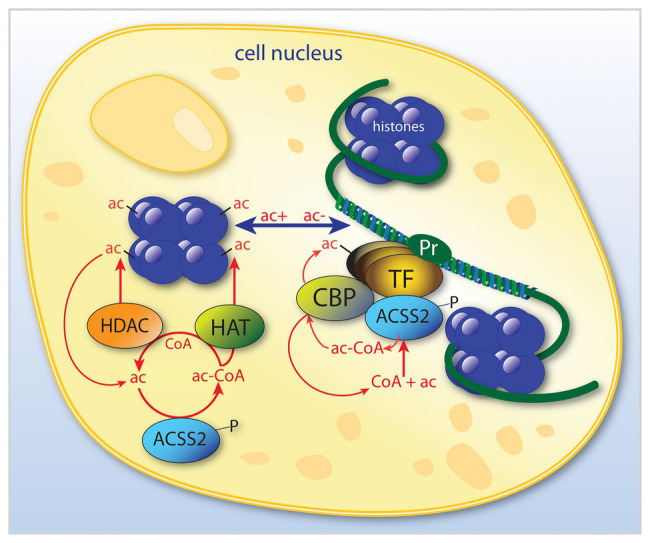
Simplified schematic of the dual roles of ACSS2 in the modulation of transcription in cell nuclei. ACSS2 is involved in the modulation of selective transcription factors by at least two mechanisms. By acting to selectively acetylate (ac) certain histones, ACSS2 facilitates opening of nucleosomes (shown in purple) at specific gene promoter regions (Pr). Further, ACSS2 is also involved in enhancing transcription of those genes *via* acetylation of transcription factor complexes. ACSS2 acts in the nucleus by additional acetylation mechanisms, for example, many nuclear-localized enzymes are activated or deactivated by ACSS2-facilitated acetylation, including ACSS2 itself, which is inactivated by acetylation. ac, acetate; ac-CoA, acetyl-CoA; ACSS2, acyl-CoA short chain synthetase family member 2; CBP, CREB binding protein; HAT, histone acetyltransferase; HDAC, histone deacetylase; P, phosphorylation site on ACSS2; Pr, gene promoter; and TF, transcription factor.

ACSS2 is present in the cytoplasm and nucleus of most cell types. However, it is expressed at much higher levels in cell nuclei in the rat brain (Ariyannur et al., 2010; Moffett et al., 2013). The strong nuclear localization of ACSS2 in many cell types has often been overlooked, in part due to the role of ACSS2 in cytoplasmic lipid synthesis. Importantly, ACSS2 has been shown to translocate from the cytoplasm to the nucleus (Chen et al., 2017), which will be discussed in more detail below. This type of translocation is associated with hormonal and transcription factor signaling from the cell periphery to the cell nucleus to induce or enhance the transcription of specific genes. Mounting evidence indicates that this applies to ACSS2. It is clear that ACSS2 is involved in a number of regulatory roles affecting gene transcription, as is the case with other enzymes that can be recruited to transcription factor complexes. However, ACSS2 is unique in that it is the only known enzyme capable of utilizing free acetate in cell nuclei. This places ACSS2 in a position to provide localized acetyl-CoA in response to any local increase in acetate levels within the nucleus. As such, ACSS2 helps fuel all acetyltransferases that operate in the cell nucleus. By providing a local supply of acetyl-CoA, ACSS2 facilitates all acetylation reactions in the nucleus. Therefore, ACSS2 can influence gene transcription through two critically important types of acetylation reactions; the acetylation of specific of histones and transcription factors (Figure 3). It is in these two roles of facilitating histone and transcription factor acetylation that ACSS2 can affect metabolic reprograming and cell cycle progression in cancer cells through epigenetic means.

In order to better understand the roles of ACSS2 in cancer, it is helpful to compare its function to that of ATP-citrate lyase (ACLY). Homozygous *ACLY* knockout is embryonic lethal (Beigneux et al., 2004), whereas *ACSS2* knockout is not (Comerford et al., 2014), indicating that in mammals ACSS2 cannot substitute for ACLY in supplying all requisite cytoplasmic and nuclear acetyl-CoA. Intriguingly, adult *ACSS2* knockout mice appear phenotypically normal, however, they exhibit reduced tumor burdens in models of hepatic cancer (Huang et al., 2018). Paradoxically, in other studies decreased expression of ACSS2 promotes metastasis and predicts poor prognosis in hepatocellular carcinoma (Sun et al., 2017). In renal cell carcinoma, *ACSS2* knockdown inhibited growth, migration, and invasion, whereas overexpression of ACSS2 enhanced these effects, implying that ACSS2 increases renal cell tumor aggressiveness (Zhang et al., 2018). Reconciling these discrepancies will be critical for understanding the actions associated with ACSS2 in cancer cells.

Studies on acetate utilization *via* ACSS2 in cancer have shown that acetate is released by cancer cells under hypoxic conditions, and that inhibition of ACSS2 reduces acetate production and release (Yoshii et al., 2009a). The authors proposed that ACSS2 in cancer cells is capable of running the reverse reaction and generating acetate from existing acetyl-CoA. These investigators also reported that tumor cells take up and metabolize acetate to lipids (Yoshii et al., 2009b). Such studies emphasize the traditional role of ACSS2 in lipid synthesis and suggest that acetate supports biomass accumulation in cancer cells under hypoxic conditions. The authors hypothesize that ACSS2 may act to buffer acetate and acetyl-CoA levels in cancer cells (Yoshii et al., 2015). Acetate utilization has also been documented in glioblastoma (grade IV astrocytoma), the most common adult primary brain tumor. Using human orthotopic tumors in mice and infusions of ^13^C-labeled glucose, acetate, and glutamine, Mashimo et al. (2014) showed that glioblastoma cells oxidize both glucose and acetate, but not glutamine. They found that the ratio of glucose to acetate utilization in the citric acid cycle of normal mouse brain tissue was over 5:1 (85% glucose vs. 15% acetate). In orthotopic glioblastoma tumors, the ratio shifted to approximately equal oxidation of both substrates. They also noted that the increase in acetate utilization can be explained in part by the fact that glioblastoma cells are derived from astrocytes, which are known to avidly take up and oxidize acetate (Waniewski and Martin, 1998). Further, they proposed that ACSS2 was responsible for the increased acetate oxidation by glioblastoma cells, even though acetyl-CoA generated in the cytoplasm and nucleus cannot gain access to the mitochondrial matrix, where acetate oxidation occurs. Studies with *ACSS2* knockout mice confirmed earlier work showing that the enzyme was involved in both lipid synthesis and histone acetylation. Comerford et al. (2014) also showed that *ACSS2* knockout mice had reduced tumor burden in two models of hepatocellular carcinoma, indicating that acetate conversion to acetyl-CoA by ACSS2 was an important factor in promoting tumor growth in this cancer type. Paradoxically, Hur et al. (2015) showed that the loss of ACSS2 expression in gastric cancer correlated with poor survival and outcomes. Similarly, loss of ACSS2 expression was associated with increased aggressiveness and progression of colorectal cancer (Bae et al., 2016). Clearly, ACSS2 can have contradictory actions in different tumor types, further highlighting the fact that its actions in oncogenesis and tumor progression involve more than lipid synthesis.

In a fascinating series of experiments using *Acss2* knockout mice, Huang et al. (2018) noted that the enzyme was involved in the selective regulation of lipid metabolism. When fed a high-fat diet, *Acss2* knockout mice did not become as obese as wild-type mice and did not develop hepatic steatosis like the wild-type mice. When fasted, *Acss2*−/− mice lost weight faster, were weaker, and had lower locomotor activity than fasted *Acss2*+/+ mice. Further, in the fasted knockout mice, serum glucose and ketone bodies were reduced, whereas serum non-esterified fatty acid levels were increased. Indeed, *Acss2* knockout had wide-ranging effects on nutrient uptake, metabolism and utilization that were tissue-specific. Huang et al. noted that ACSS2 acts more like a transcription factor than a metabolic enzyme, whereby it initiates dynamic reprograming of gene expression in various tissues to coordinate the proper physiological adaptation of animals to the fed or fasted state. In this same context, ACSS2 can reprogram cancer cells to adapt to nutrient deprivation and other stressors, such as hypoxia.

### ACSS2 in Nuclear Acetate Recycling

Our early immunohistochemical studies into the localization of ACSS2 surprisingly demonstrated that expression in the rat brain was predominantly nuclear, rather than cytoplasmic (Ariyannur et al., 2010). In the first detailed examination of ACSS2 expression in the brain, we observed nuclear expression in all major cell types including neurons, oligodendrocytes, and astrocytes. In normal adult rats, ACSS2 expression was modest and was present in a relatively small number of cell nuclei throughout the brain. It was also present in the cytoplasm, nucleus, and axons of select neurons in the brainstem, cranial nerve ganglia, and spinal cord. In contrast, many more cells strongly expressed ACSS2 in their nuclei during postnatal brain development and in response to experimental brain injury (discussed in part 1 of this review). The large increase in the number of cells with nuclear ACSS2 expression during development and in response to injury strongly implicated ACSS2 in nuclear acetylation reactions and transcriptional regulation required for cellular differentiation, stress responses, and repair (Ariyannur et al., 2010; Moffett et al., 2013). More recent studies have shown that in cancer cells, ACSS2 is expressed in cell nuclei, where it operates to recycle acetate derived from HDAC-mediated deacetylation reactions during hypoxia (Bulusu et al., 2017). Similar results have been obtained in studies of spermatogenic cells, wherein ACSS2 was localized to the nucleus and acted to recycle acetate derived from HDAC reactions (Goudarzi and Amiri-Yekta, 2020).

Hypoxia is often associated with the tumor microenvironment, and tumor tissue responds by activation of the transcription factor hypoxia inducible factor (HIF) (reviewed in Ratcliffe, 2013). The HIF transcriptional control system is complicated and regulates a wide array of signaling pathways. The functional roles of acetate in cancer cell proliferation remain to be determined, yet it is clear that they are dependent on the cell type from which the cancer was derived (Pandey et al., 2018b). While acetate could promote energy derivation *via* ACSS1, or lipogenesis for biomass accumulation *via* ACSS2, it could also act to regulate metabolic pathways by shifting the pattern of histone and transcription factor acetylation. For example, it has been found that HIF-2α acetylation is an important regulator of erythropoietin expression under hypoxic conditions (Xu et al., 2014; Chen et al., 2015). Erythropoietin is synthesized extensively in the kidney and liver of adult mammals and is involved in controlling erythrocyte production. Erythropoietin synthesis is regulated by the stress-responsive transcription factor HIF-2. Effective HIF-2 dependent induction of erythropoietin expression during hypoxia requires acetylation of the HIF-2α subunit *via* the lysine acetyltransferase CREB-binding protein (CBP; Xu et al., 2014; Chen et al., 2015). These studies further showed that, in acutely anemic mice, ACSS2 was required in conjunction with CBP for HIF-2α acetylation, CBP-HIF-2α complex formation, CBP-HIF-2α recruitment to the erythropoietin enhancer, and efficient induction of erythropoietin gene expression. Furthermore, acetate supplementation with GTA augmented stress erythropoiesis in an ACSS2-dependent manner in acutely anemic mice. Similarly, in cancer cells exposed to glucose or oxygen deprivation, there is an increase in intracellular acetate levels and translocation of ACSS2 from the cytoplasm to the nucleus. Once in the nucleus, ACSS2 acetylates HIF-2α *via* the coactivator CPB, leading to changes in histone acetylation and promoting *de novo* transcription (Chen et al., 2017). These studies emphasized the regulatory role of transcription factor acetylation in the control of erythropoietin gene expression during pathophysiological states marked by tissue hypoxia, and the key role played by ACSS2 in mediating this response. In studies of hepatocellular carcinoma, *ACSS2* knockdown was found to deacetylate HIF-2α, which, in turn, increased cell migration and invasion and induced epithelial-mesenchymal transition (Sun et al., 2017). Li et al. (2017) reported that nuclear localized ACSS2 promotes lysosomal biogenesis, autophagy and brain tumorigenesis. Using human glioblastoma cells, they showed that glucose deprivation led to AMPK phosphorylation of ACSS2 at serine 659 (ACSS2-pS659), which exposes a nuclear localization signal leading to nuclear import. They also found that ACSS2 associates with transcription factor EB (TFEB) and binds to promoter regions for genes associated with lysosomal biogenesis and autophagy. These investigators posit that nutrient deprivation in the tumor microenvironment elicits nuclear translocation of ACSS2 to provide an efficient method for reconverting the acetate generated from deacetylation reactions to acetyl-CoA for use in re-acetylating histones in subsequent chromatin remodeling events (Li et al., 2017). It is important to note here that NAT8L, the enzyme that synthesizes NAA (also known as aspartate N-acetyltransferase), has also been associated with nuclear translocation of TFEB and induction of autophagy (Huber et al., 2019). This will be discussed in more detail below in the context of NAA signaling.

In other studies, the expression levels of ACSS2-pS659 were analyzed by immunohistochemistry in surgical specimens from 303 non-small-cell lung carcinoma patients (Yang et al., 2019). Increased expression of ACSS2-pS659 in tumors as compared with normal surrounding tissue was identified as a reliable prognostic marker for patients with poor survival rates. In another study of esophageal squamous carcinoma cells in culture, the cancer cells were found to be less sensitive to nutrient deprivation than normal cells. Further, ACSS2 was upregulated in the cancer cells and inhibition of ACSS2 *via* siRNA greatly reduced cancer cell proliferation under nutrient stress, indicating the importance for acetate utilization through ACSS2 in these cells (Mi et al., 2019).

Recently, two alternative transcription start sites for the ACSS2 gene have been identified in hepatocellular carcinoma cells that may help explain the dual role of ACSS2 in promoting and inhibiting cancer aggressiveness (Wang et al., 2019). Alternative transcription start site selection produced two different ACSS2 transcripts, ACSS2-S1 and ACSS2-S2. The two isoforms of ACSS2 had different subcellular localization and action. The ACSS2-S1 isoform was expressed predominantly in the cytoplasm, whereas the ACSS2-S2 isoform was distributed in both the nucleus and cytoplasm. Overexpression of ACSS2-S2 promoted cell proliferation and invasion, whereas overexpression of ACSS2-S1 did not. Such findings indicate that ACSS2 has a broader functional repertoire than acting as a salvage pathway for reclaiming acetate, and more than a parallel pathway to ACLY for cytoplasmic acetyl-CoA production. We suggest that ACSS2 is an important modulator of the integrated stress response (ISR) in many cell types, including acting as a transcriptional modulator in cellular responses to infection and injury.

ACSS2 acts as a specific regulatory pathway for protein acetylation that is distinct from the actions of ACLY. If ACSS2 associates specifically with certain transcription factors and provides the necessary acetyl-CoA in a local on-demand basis, then it may be a key regulatory agent in epigenetic control of many cell functions associated with nutrient or oxygen deprivation. It is likely that ACSS2 acts to amplify the activity of a wide range of regulatory pathways. However, acetate and ACSS2 are involved in numerous additional regulatory functions other than responding to nutrient and oxygen deprivation. For example, both the substrate and enzyme are involved in regulatory actions in monocyte-derived dendritic cells in response to signals including zymosan and lipopolysaccharide. Results suggested that when acetyl-CoA formation from pyruvate was low, ACSS2 replenished the cytoplasmic and nuclear supplies, thus supporting cytokine transcription (Marquez et al., 2019). While one major acetate source providing substrate for ACSS2 is through ubiquitous protein deacetylation reactions, such as histone deacetylation, NAA is another readily available source of acetate in cancer cells that express NAT8L, ASPA, and ACSS2. This is where we turn our attention now.

### The “NAA Signaling Pathway” in Cancer

The acetylated form of aspartate, NAA, was long thought to be synthesized exclusively in the brain (Tallan, 1957; Benuck and D’Adamo, 1968; Miyake et al., 1981), where it is the most highly concentrated acetylated metabolite (reviewed in Moffett et al., 2007). In early studies, the levels of NAA in several peripheral tissues had been reported to be extremely low (Miyake et al., 1981), and it was concluded that NAA was only synthesized in the nervous system. This conclusion was overturned when increased NAA levels were found in ovarian cancers (Kolwijck et al., 2009, 2010; Fong et al., 2011). In 2013, Pessentheiner et al. demonstrated that NAA was synthesized and utilized extensively in adipose tissue for energy derivation and lipid turnover (Pessentheiner et al., 2013), indicating both synthesis and functional roles for NAA outside the nervous system (Prokesch et al., 2016; Bogner-Strauss, 2017; Huber et al., 2019). Also in 2013, we showed that NAA promoted growth and inhibited differentiation of tumor derived glioma stem-like cells in culture (Long et al., 2013a), implicating NAA in cancer growth. Shortly, thereafter a number of studies demonstrated that NAA was present in high concentrations in certain cancer cells, including breast and lung cancer, but not in the surrounding normal tissues (Terunuma et al., 2014; Lou et al., 2016; Weindl et al., 2016; Zand et al., 2016). The biosynthetic and degradative enzymes of NAA metabolism, aspartate N-acetyltransferase (Asp-NAT; gene *NAT8L*), and ASPA respectively, have now been detected in a number of cancer cell types, indicating that local NAA synthesis and metabolism were possible in certain cancers despite the fact that the tissues of origin for the cancers do not normally synthesize NAA.

Metabolic profiling in high grade serous ovarian cancer and normal ovarian tissue showed that patients with high NAA levels and *NAT8L* mRNA expression had worse overall survival than patients with low NAA levels and decreased *NAT8L* expression (Zand et al., 2016). Higher *NAT8L* expression in other cancers (melanoma, renal cell, breast, colon, and uterine) also was associated with overall worse survival. Silencing of *NAT8L* reduced cancer cell viability and proliferation *in vitro* in multiple cancer cell lines. This effect was reversed by addition of exogenous NAA. In orthotopic mouse models of melanoma and ovarian cancer, *NAT8L* silencing reduced cancer growth. Further, *NAT8L* silencing downregulated anti-apoptotic pathways mediated by the transcription factor FOXM1 (Zand et al., 2016).

NAA has also been shown to play a role in lung cancer (Lou et al., 2016). High concentrations of NAA were detected in several non-small cell lung cancer (NSCLC) cell lines as well as in NSCLC patient tumors. Further, examination of the Cancer Genome Atlas revealed that elevated *NAT8L* expression was detected in approximately 40% of adrenocarcinoma and squamous cell carcinoma cases (*N* = 577) relative to expression in all non-malignant lung tissue (*N* = 74). The authors also showed that siRNA mediated suppression of *NAT8L* in NSCLC cells reduced intracellular NAA levels and NAA secretion. Importantly, they showed that NAA synthesis in NSCLC cells was dependent upon the supply of glutamine, linking NAA synthesis to the increased glutaminolysis observed in many cancers.

In a study by Wynn et al. (2016), the oncogene RhoC was found to be a regulator of glutamine metabolism and NAA production in cell lines of inflammatory breast cancer (IBC), an extremely lethal cancer that rapidly metastasizes. Using metabolic assays, including ^13^C tracer experiments, it was found that SUM149 cells, the primary *in vitro* model of IBC, exhibit metabolic abnormalities including increased glutamine uptake and synthesis of NAA. These cells showed low TCA cycle activity from glucose and very low citrate synthesis from labeled pyruvate. When NAA synthesis was examined, the mass labeled NAA was not derived from labeled glucose, but rather was derived from labeled glutamine. *RhoC* knockdown reduced glutamine consumption and NAA levels. We hypothesize that the connection between glutamine and NAA synthesis in IBC cells may be associated with the Warburg effect (aerobic glycolysis) and may contribute to the rapid proliferative behavior of IBC cells.

A study involving non-targeted stable isotope labeling and metabolic analysis showed that metabolic flux changes occurred in response to decreased oxygen availability in human adrenocarcinoma cells, leading to an increased glutamine contribution to acetyl-CoA production (Weindl et al., 2016). These investigations also revealed biosynthesis of NAA *via* NAT8L in lung cancer cells and showed that NAT8L silencing inhibits proliferation of several different lung cancer cell lines.

**Figure 4:**
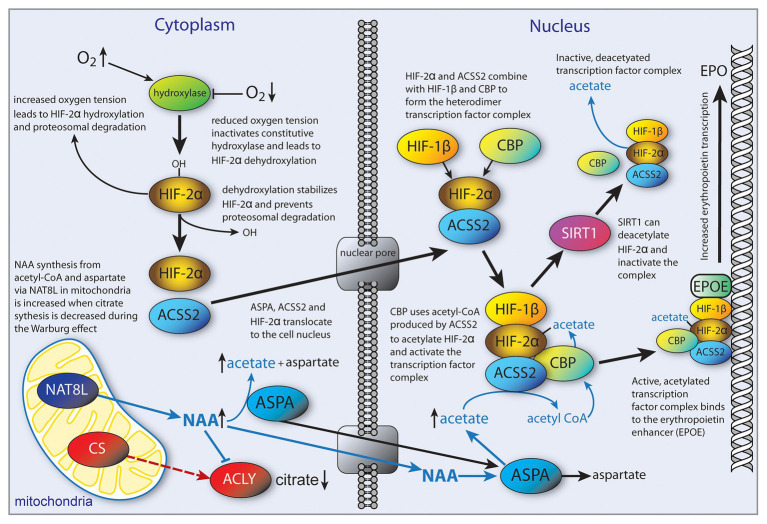
Hypothesized role of NAA in cancer cells as an alternative source of acetyl-CoA for specific transcription factor acetylation in place of citrate. We propose that some transcription factors use the NAT8L-ASPA-ACSS2 pathway (“NAA pathway”) preferentially over the citrate-ACLY pathway. Activation of the hypoxia inducible factor-2 alpha (HIF-2α) transcription factor during hypoxia is shown as an example. Citrate synthesis is reduced due to the Warburg effect (possibly by direct NAA inhibition of ACLY) and NAA can act as an alternative acetyl-CoA source by the sequential actions of ASPA and ACSS2 (orange pathway). Both ASPA and ACSS2 have been shown to shuttle between the cytoplasm and nucleus. This general mechanism could be involved in a number of transcription factor signaling pathways. For example, transcription factor EB (TFEB), which controls autophagy, is responsive to NAA. See figure and text for additional details. ACLY, ATP citrate lyase; ACSS2, acyl-CoA short chain synthetase family member 2; ASPA, aspartoacylase; CBP, CREB binding protein (acetyltransferase); CS, citrate synthase; EPO, erythropoietin gene; EPOE, erythropoietin enhancer; HIF, hypoxia inducible factor; NAA, N-acetylaspartate; NAT8L, aspartate N-acetyltransferase (NAA synthase); and SIRT1, sirtuin-1 (deacetylase). Figure adapted from Jaworski et al. (2016).

**Figure 5:**
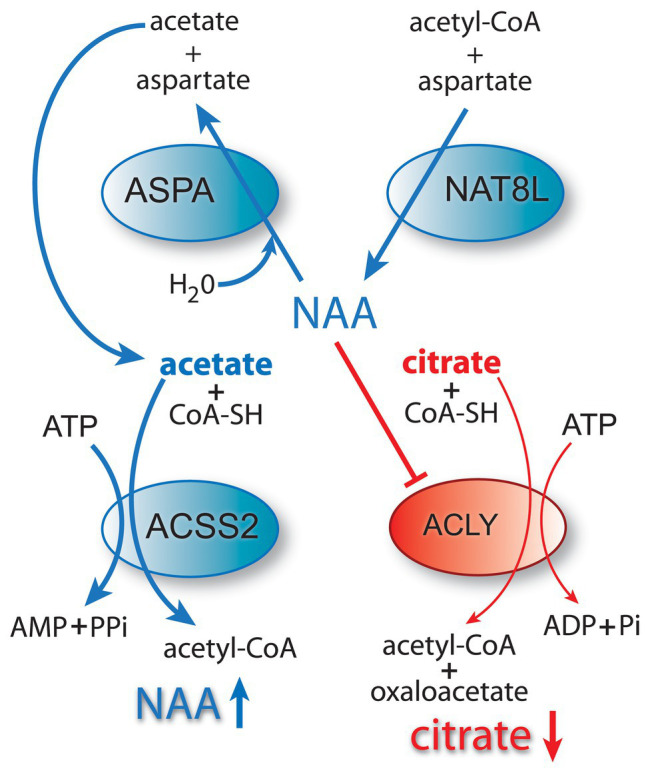
The NAA and citrate pathways to acetyl-CoA. The NAA-ASPA-ACSS2 pathway to acetyl-CoA formation is shown in blue, whereas the citrate lyase pathway is shown in red. High NAA levels inhibit ACLY activity in brown adipose tissue, and this may turn out to be a more general phenomenon in other cell types including oligodendrocytes and certain types of cancer cells. This would shift mitochondrial output of the tricarboxylic acid cycle from citrate to NAA, which would in turn activate the ACSS2 pathway to acetyl-CoA formation. When this pathway is activated in the cell nucleus, it can affect the activity of select transcription factors and modulate transcriptional activity.

**Figure 6:**
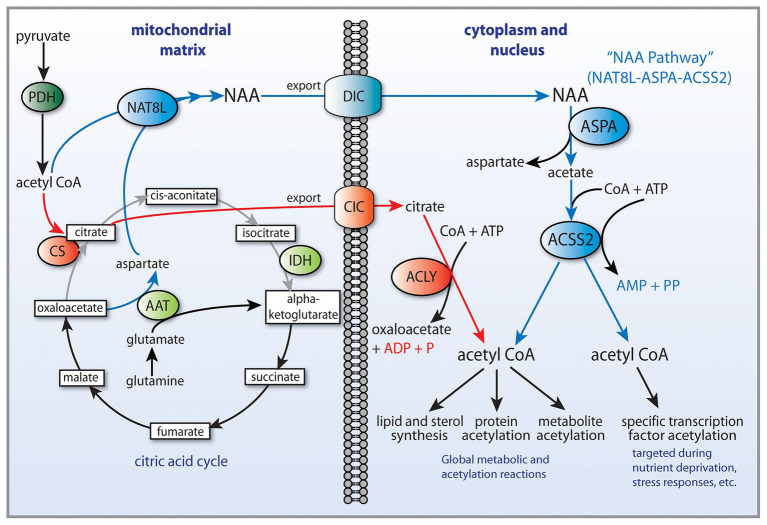
NAA as a selective, alternative cytoplasmic and nuclear acetyl-CoA source. Reduced citrate synthase activity in cancer cells may result in increased NAA synthesis and export from mitochondria. Citrate (red pathway) and NAA (blue pathway) are synthesized from acetyl-CoA in cancer cell mitochondria. Both citrate and NAA are exported from mitochondria to the cytoplasm. The transporter that moves NAA out of the mitochondrial matrix is not known, but the most likely candidate is the dicarboxylate transporter (DIC). Citrate is converted in the cytoplasm into acetyl-CoA, which can then be used for lipid synthesis as well as protein acetylation reactions in the cytoplasm and nucleus. NAA represents an alternative pathway that may allow for transport of acetate groups out of mitochondria, especially under hypoxic conditions when citrate synthase activity is reduced. The portion of the citric acid cycle between citrate and alpha ketoglutarate is reduced (gray lines), and the enzyme aspartate aminotransferase supplies alpha-ketoglutarate from glutamine *via* glutamate (glutaminolysis). After synthesis, NAA is transported to the cytoplasm and is converted to acetate and aspartate by ASPA. Subsequently, acetate plus CoA are converted to acetyl-CoA by ACSS2. This may represent a salvage pathway for delivering acetyl-CoA from cancer cell mitochondria under hypoxic conditions, when citric acid cycle and citrate synthase activities are reduced. Additionally, ACSS2 provides for a pathway to specific transcription factor acetylation by its selective complex formation with certain transcription factor complexes such as hypoxia inducible factor 2α (Chen et al., 2015). AAT, aspartate aminotransferase; ACSS2, acyl coenzyme A synthetase short-chain family member 2; ACLY, ATP-citrate lyase; ASPA, aspartoacylase; CS, citrate synthase; CIC, citrate carrier; DIC, dicarboxylate transporter (SCL25A10); IDH, isocitrate dehydrogenase; PDH, pyruvate dehydrogenase; and NAT8L, aspartate N-acetyltransferase (aka NAA synthase).

The source of acetyl-CoA for protein acetylation, including histone acetylation, has been assumed to be citrate exported from mitochondria, but acetyl-CoA derived from acetate *via* ACSS2 has been implicated as an alternative source (Takahashi et al., 2006; Wellen et al., 2009; Ariyannur et al., 2010; Moffett et al., 2013). As discussed above, findings on the transcription factor HIF-2α provided a mechanism by which specificity could be maintained in the signaling pathway through the action of ACSS2. Chen et al. showed that ACSS2 and CREB binding protein are both required for HIF-2α acetylation and recruitment to the erythropoietin promoter (Chen et al., 2015). The acetylation was specific for ACSS2, indicating that ACSS2 forms part of the transcription factor complex, and thus the acetyl-CoA derived from free acetate would be distinct from the acetyl-CoA formed from citrate (reviewed in Jaworski et al., 2016). NAA can act as an acetyl group donor for certain protein acetylation reactions by the sequential action of ASPA and ACSS2 (Figures 4–6; Moffett et al., 2013). Several studies have shown that ASPA is difficult to extract from nuclear fractions under heat/high salt conditions, suggesting that nuclear ASPA is tightly associated with chromatin (Hershfield et al., 2006; Long et al., 2013b). This implies that ASPA is also incorporated into specific transcription factor complexes, as is the case with ACSS2.

We hypothesize that the NAA-ASPA-ACSS2 pathway may provide acetyl-CoA for the acetylation of distinct groups of transcription factors that are activated under different physiological conditions such as hypoxia and nutrient deprivation (Figure 4). Recently, NAA has been shown to have actions in the nucleus, whereby it alters histone H3 methylation status, providing further evidence that NAA may be involved in chromatin remodeling associated with gene transcription (Singhal et al., 2016). We propose that NAA provides a unique source of acetate for intrinsic acetyl-CoA production within transcription factor complexes, especially in certain cancer cells, where oxidative phosphorylation and citrate production are often reduced. It is therefore useful to differentiate these disparate routes of acetyl-CoA synthesis as the citrate-acetyl-CoA and NAA-acetyl-CoA pathways (Figure 5).

Studies by Prokesch et al. in brown and white adipose tissue have been very instructive in demonstrating the linkages between NAA, energy homeostasis, lipid turnover, and histone acetylation. One important finding in these studies was that reduced ASPA activity and increased NAA levels inhibit *ACLY* mRNA expression in an additive manner (Prokesch et al., 2016; Hofer et al., 2019), indicating cross-talk between the NAA and citrate synthetic systems (Figure 5). Because citrate and NAA are both export products of mitochondrial metabolism, this type of cross-inhibitory mechanism would greatly favor NAA synthesis and export over that of citrate when NAA levels were high and ASPA activity was low. The same studies demonstrated several additional aspects of increased NAA levels including reduced expression of transcription factors associated with brown adipocyte differentiation (Prokesch et al., 2016), linking high NAA levels to inhibition of cell maturation. Further, ASPA and ACSS2 expression were also downregulated by high intracellular NAA concentrations and this was associated with reduced H3K9 and H3K27 acetylation. It is unclear if these effects of NAA on protein expression and histone acetylation are specific to adipose cells or if they pertain to other cell types including cancer cells. However, mounting evidence suggests that some of the effects of excessive NAA concentrations may impact the function of many cell types, especially cancer cells. If so, the question arises as to whether the effects of NAA on cell behavior are direct, or indirect through the generation of acetyl-CoA by the sequential action of ASPA and ACSS2. In the case of high levels of NAA leading to reduced expression of ACLY in adipocytes, the effect is most likely through the NAA signaling pathway. If the effects of high NAA levels are found to be direct in some proteins, then certain core metabolic enzymes may have regulatory binding sites for NAA that modulate protein function. It is also possible that NAA could directly affect other key regulatory enzymes such as HDACs, which would then impact histone acetylation levels directly. As noted above, in Canavan disease where ASPA activity is absent and NAA levels are high, histone H3 acetylation is increased in oligodendrocytes (Kumar et al., 2009; Mattan et al., 2010), including at loci such as H3K9 (Moffett et al., 2014). This would suggest that at high concentrations, NAA could act as an HDAC inhibitor, thus increasing acetylation at specific sites. However, in brown adipocytes, Prokesch et al. (2016) found that decreased ASPA expression and increased NAA concentrations led to reduced histone acetylation levels, suggesting that NAA could act as an HDAC enhancer. They noted that the effect appeared to be direct, rather than through acetyl-CoA, because cytoplasmic acetyl-CoA levels in NAA-supplemented cells were unchanged. The opposite effects on histone acetylation levels in different cell types could be due to the expression of different HDACs in oligodendrocytes and brown adipocytes, which could be differentially sensitive to high concentrations of NAA. This is a potentially fruitful research topic that could lead to discoveries about the links between NAA signaling and altered cell behavior in some cancers.

The evidence favors the view that ACLY provides robust, but relatively non-targeted acetyl-CoA production for all acetyl-CoA pools, whereas the NAA-ASPA-ACSS2 system provides a less quantitative, but more targeted, acetyl-CoA pool through protein-complex formation, including transcription factor complexes. We propose that one purpose of the NAA-ASPA-ACSS2 system is to preferentially target NAA output from mitochondria to lipid synthesis and acetylation reactions that are, in some cases, more restricted and local than those served by the larger citrate-ACLY pathway for generating nuclear and cytoplasmic acetyl-CoA pools (Figure 6). We further propose that this alternate, selective pathway be designated as the NAT8L-ASPA-ACSS2 signaling pathway, where the acronym would be the “NAA” signaling pathway.

If these observations are confirmed and extended to cancer, then NAA metabolism may be an important mechanism for exporting an acetyl-CoA precursor from mitochondria in cancer cells under hypoxic conditions, and may, in part, help explain certain aspects of the Warburg effect and its more recent variation, the “reverse Warburg effect.” This revision of the Warburg effect hypothesis posits that different cell types within a tumor utilize different and complementary energy substrates and that the interplay between the distinct cell types drives tumor progression (Pavlides et al., 2009; Potter et al., 2016). In this hypothesis, stromal cells (e.g., fibroblasts and pericytes) and epithelial cancer cells are metabolically distinct, yet coupled. The stromal cells employ glycolysis and produce lactate and other substrates including ketone bodies and pass these to the epithelial cancer cells, which utilize them for oxidative phosphorylation (Martinez-Outschoorn et al., 2012). It is thought that the epithelial cancer cells secrete reactive oxygen species, which drives autophagy and glycolysis in the stromal fibroblasts (Pavlides et al., 2012). In this scenario, the tumor fibroblasts exhibit the Warburg effect and excrete energy-deriving metabolites, whereas the epithelial cancer cells employ the reverse Warburg effect and utilize these energy substrates for oxidative phosphorylation. It is not clear what cell types produce and release NAA in different cancers, but it is likely that the cellular source of NAA varies between tumor types, dependent upon the cells of origin and the specific metabolic reprograming involved in oncogenesis in those cell types. In several *in vitro* studies, NAA has been shown to be produced by the cancer cells themselves, including NSCLC cells (Lou et al., 2016) and inflammatory breast cancer cells (Wynn et al., 2016). If the NAA is released by cancer cells and is taken up by neighboring cancer or stromal cells for metabolism, then NAA would be an energy-deriving metabolite that did not fit the reverse Warburg effect role. It is noteworthy that the related dipeptide, N-acetylaspartylglutamate (NAAG), which is synthesized enzymatically from NAA and glutamate, has also been linked to cancer cell proliferation (Long et al., 2013a; Nguyen et al., 2019).

Speculating on the origins of the NAA signaling pathway for targeted acetyl-CoA generation, it is plausible to conclude that this system evolved first in adipocytes, where all of the enzymes (NAT8L, ASPA, and ACSS2) are coexpressed in a single cell type (Pessentheiner et al., 2013; Prokesch et al., 2016). In this situation, NAA acts very similar to the citrate system as a method of transferring an acetyl-CoA product to the cytoplasm, where acetyl-CoA can then be regenerated. In the nervous system, a more complicated system evolved, wherein the synthetic (NAT8L) and degradative (ASPA) compartments were largely segregated into different cellular populations. NAA synthesis occurs in neurons and the NAA is transferred to oligodendrocytes (Chakraborty et al., 2001), where ASPA and ACSS2 are expressed (Ariyannur et al., 2010; Moffett et al., 2011). The NAA derived acetate liberated in oligodendrocytes can go on to participate in lipid synthesis or protein acetylation reactions. In this expanded role including intercellular transport, NAA takes on a new role that is trophic in nature (Moffett et al., 2014). It is in this new role that NAA may play a significant role in the progression of some cancers.

If excess NAA promotes growth and inhibits differentiation in other cell types, as it does in the case of glioma stem-like cells (Long et al., 2013a), then NAA may turn out to be another one of the driving forces in oncogenesis for certain tumor types. Sustained NAA synthesis in the absence of sufficient ASPA activity would lead to increasing intracellular levels, and this could help drive proliferation while also inhibiting differentiation. However, this is clearly not a universal effect of NAA on cells. Mazzoccoli et al. (2016) found that the SH-SY5Y neuroblastoma cell line responded to NAA treatment with the opposite behavior; that of reducing growth and inducing differentiation. This implies that NAA has opposing signaling functions in neuroblastoma and glioma stem-like cells. The SH-SY5Y neuroblastoma cell line is well-characterized and synthesizes both NAA and NAAG (Arun et al., 2004, 2006, 2008, 2009, 2010c). Glioma stem-like cells, in contrast, are of astrocytic origin and astrocytes do not normally synthesize NAA or NAAG. As such, NAA has signaling functions that lead neural-derived cell lines to differentiate but cause astrocyte-derived cell lines to proliferate. These types of opposing effects in distinct cell types are consistent with NAA having a role in modulating cell behavior through the action at specific transcription factor complexes. The data are also consistent with selective activation of enzymes and receptors though targeted acetylation. It is not known if NAA also has direct signaling activity through receptors, but to date, no receptor has been confirmed to be activated by physiological levels of NAA. One earlier report that NAA activated G protein-coupled metabotropic glutamate receptors in hippocampal neurons (Yan et al., 2003) has not been confirmed to our knowledge.

Some of the more intriguing lines of evidence demonstrating signaling functions for NAA come from studies on *NAT8L* knockout mice and NAA supplementation. In studies with whole-body (*NAT8L*-KO) and adipocyte-specific (*NAT8L*-AKO) knockout mice, Hofer et al. (2019) found that NAA is linked to glucose, lipid, and energy metabolism. In *NAT8L*-KO mice they found increased food intake, increased energy expenditure (kcal/min), increased glucose-stimulated insulin secretion, and reduced blood glucose levels during glucose tolerance tests, relative to wild type mice. Further, in adipocytes from the *NAT8L*-KO mice, ATP levels and oxygen consumption rates were increased relative to wild type mice, and the expression of uncoupling protein-1 (UCP-1) was significantly upregulated. Importantly, within 2 h of administering 400 nM NAA to MIN6 mouse insulinoma cells in culture, glucose stimulated insulin secretion was significantly reduced (Hofer et al., 2019). This effect on insulin secretion is too rapid to involve gene transcription, strongly suggesting that NAA has other signaling functions related to energy homeostasis. Further, MIN6 cells are of pancreatic origin, indicating that NAA has signaling functions outside the nervous system and adipose tissues. The mechanisms whereby NAA can influence energy derivation clearly involve mitochondrial metabolism and links to the TCA cycle, but effects on insulin secretion in target cells that do not synthesize NAA are more difficult to explain currently. Based on investigations into Canavan disease, we have proposed that NAA may have direct signaling functions (Appu et al., 2017). These could include activation or inhibition of enzymes, receptors, and other proteins *via* acetyl-transferase-mediated acetylation or direct actions at as yet unidentified receptors.

In summary, the NAA signaling pathway has several arms including (1) local chromatin remodeling through selective histone acetylation *via* acetyl-CoA and acetyltransferases such as p300, (2) transcriptional effects through acetylation of specific transcription factors *via* acetyl-CoA, and (3) direct action of NAA on as yet unidentified protein targets, possibly including enzymes as in the case of ACLY, as well as uncharacterized receptors. While the NAA signaling pathway that we have proposed is hypothetical, it can explain how NAA-derived acetate affects gene transcription. Whether this pathway operates in organ systems other than the nervous system and adipose tissue also remains to be determined. But the role this pathway appears to play in many cancers suggests that the NAA pathway may be involved in regulation of transcription in a number of different tissues in response to physiological stress.

### Glyceryl Triacetate and Cancer

Acetate has received substantial attention as a prominent source of acetyl-CoA for cancer biomass accumulation. Acetate has been shown to promote cancer cell proliferation (Kamphorst et al., 2014) and to act as an epigenetic signaling agent to promote lipid synthesis during hypoxia (Gao et al., 2016). However, the effects of exogenously supplied acetate or acetate precursors on cancer cells have provided conflicting results depending upon factors such as the cancer cell type and the molecular source of the acetate. As noted in the previous section, NAA provides a unique source of acetate that promotes growth in some cancer cell types and inhibits growth in others. GTA has been found to have actions on cancer cell growth that are distinct in some respects to the effects of acetate alone (reviewed in Jaworski et al., 2016). GTA was found to induce cytostatic G_0_ growth arrest of oligodendroglioma and astrocytoma derived cells *in vitro*, without affecting the growth of normal cells (Long et al., 2013b, 2015).

Sodium acetate, at doses comparable to that generated by complete GTA hydrolysis, also promoted growth arrest. Interestingly, whereas the short-chain triglyceride GTA induced growth arrest, long-chain triglycerides promoted cell growth emphasizing that fatty acid chain length can have profound effects on cancer cell behavior. In another study, acetate supplementation maintained ATP levels in glucose-deprived melanoma cells in culture, whereas other short-chain fatty acids did not (Lakhter et al., 2016). An examination of the effects of GTA on the growth of various CNS derived cell lines demonstrated that the cellular responses to GTA were dependent on cell type. GTA treatment induced G_0_ growth arrest in most oligodendroglioma-derived and glioblastoma-derived glioma stem-like cells, but increased growth of neural stem cells (Tsen et al., 2014; Long et al., 2015). Additionally, GTA was found to enhance the efficacy of the chemotherapeutic agent temozolomide in orthotopically engrafted glioblastoma and oligodendroglioma-derived glioma stem-like cells (Tsen et al., 2014). Indeed, the effects of GTA on cell growth in some cell lines were found to be dependent on what tissue culture media the cells were grown in. These findings show that acetate does not invariably support tumor cell growth, but rather, depending upon the cell type and nutrient conditions, acetate can enhance cancer cell growth, or inhibit it. The complexity of acetate metabolism and signaling functions in modulating cancer cell behavior have only been touched upon and discoveries with therapeutic potential are sure to follow.

## Conclusion and Perspective

From an evolutionary perspective, we speculate that the dual nature of ACSS2 as both a lipogenic enzyme and a regulator of stress responses, including response to nutrient deprivation, may in part be an adaptation to irregular food availability. When food is plentiful, ACSS2 can facilitate lipid synthesis and storage as is the case with ACLY. But under ketogenic conditions when food supplies are absent, ACSS2 shifts to a regulatory role to induce fatty acid oxidation and autophagy, thus helping maintain energy homeostasis. ACSS2 also functions in a regulatory fashion after other stressors including injury, hypoxia, and infection. This regulatory system may be disrupted in certain cancers, wherein ACSS2 functions abnormally to promote lipid synthesis and stress related functions, including autophagy, simultaneously. Because the role of ACSS2 is strongly dependent on cell type, its roles in the development of various cancers will be heterogeneous. Results in one type of cancer may not hold in other types, making generalizations difficult.

Acetate supplementation may find therapeutic potential in obesity, insulin resistance, diabetes, blood loss, neuroinflammation, the treatment of certain mitochondrial diseases, and as a chemotherapeutic adjuvant for specific types of cancer. ACSS1 and ACSS2 are positioned to utilize and recycle acetate in all subcellular compartments in most or all cell types. In cell nuclei, ACSS2 may be associated with an array of transcription factor complexes that enable cells to respond to hypoxia, stress, nutrient deprivation, infection, and injury, as well as other adaptive responses including memory formation. Evidence points to ACSS2 acting to modulate transcription of select genes through acetylation of selective histones and transcription factors. By directly supplying acetyl-CoA locally within the nucleus, ACSS2 acts as a transcription enhancer at select promoter regions. Acetate derived from nuclear deacetylation reactions is thus recycled by ACSS2 at active transcription sites, placing ACSS2 at a central point in acetylation-based transcriptional regulation. It is also likely that ACSS2 can modulate the activity of many other targets including enzymes, transporters, receptors, cytoskeletal proteins, etc. NAA, as a unique source of acetate and acetyl-CoA distinct from citrate, is also associated with acetate-based transcriptional regulation in certain cell types, and in many cancers.

We have presented a number of testable hypotheses including; do any central metabolic or other enzymes, for example, HDACs, have regulatory NAA binding sites that modulate their activity? Also, is the NAA signaling pathway *via* acetyl-CoA formation through ASPA and ACSS2 a bone fide regulatory pathway? If so, does it operate in tissues other than the nervous system and adipose tissue? This could be tested in several ways, for example, by determining how much NAA-derived acetate ends up bound to transcription factors and histone proteins. It would be especially important to test this in cells under physiological stress and in various cancer cell lines under conditions of hypoxia and nutrient limitation.

Understanding the mechanisms by which the NAA-ASPA-ACSS2 pathway may act to selectively regulate transcription and other cellular functions associated with acetylation will expand our knowledge of normal and neoplastic metabolic reprograming. Acetate, long discounted in human biology, is now garnering much needed attention for its roles in normal, pathological, and neoplastic cellular physiology.

## Author Contributions

JM and AN conceived the project, all authors wrote the manuscripts. All authors contributed to the article and approved the submitted version.

### Conflict of Interest

The authors declare that the research was conducted in the absence of any commercial or financial relationships that could be construed as a potential conflict of interest.
